# Healthcare providers’ adherence to breast cancer guidelines in Europe: a systematic literature review

**DOI:** 10.1007/s10549-020-05657-8

**Published:** 2020-05-06

**Authors:** Ena Niño de Guzmán, Yang Song, Pablo Alonso-Coello, Carlos Canelo-Aybar, Luciana Neamtiu, Elena Parmelli, Javier Pérez-Bracchiglione, Montserrat Rabassa, David Rigau, Zuleika Saz Parkinson, Iván Solà, Adrián Vásquez-Mejía, Ignacio Ricci-Cabello

**Affiliations:** 1 Iberoamerican Cochrane Centre - Department of Clinical Epidemiology and Public Health, Biomedical Research Institute Sant Pau (IIB Sant Pau), Sant Antonio María Claret 167, 08025 Barcelona, Spain; 2 CIBER de Epidemiología y Salud Pública (CIBERESP), Madrid, Spain; 3 European Commission, Joint Research Centre (JRC), Via E. Fermi 2749, 21027 Ispra, VA Italy; 4 Interdisciplinary Centre for Health Studies (CIESAL), Universidad de Valparaíso, Valparaíso, Chile; 5 Facultad de Medicina Humana, Universidad Nacional Mayor de San Marcos, Lima, Peru; 6 Balearic Islands Health Research Institute (IdISBa), Palma, Spain; 7 Primary Care Research Unit of Mallorca, Balearic Islands Health Service, Palma, Spain

**Keywords:** Breast neoplasms, Guidelines as topic, Evidence-based medicine, Guideline adherence, Systematic review

## Abstract

**Purpose:**

Clinical guidelines’ (CGs) adherence supports high-quality care. However, healthcare providers do not always comply with CGs recommendations. This systematic literature review aims to assess the extent of healthcare providers’ adherence to breast cancer CGs in Europe and to identify the factors that impact on healthcare providers’ adherence.

**Methods:**

We searched for systematic reviews and quantitative or qualitative primary studies in MEDLINE and Embase up to May 2019. The eligibility assessment, data extraction, and risk of bias assessment were conducted by one author and cross-checked by a second author. We conducted a narrative synthesis attending to the modality of the healthcare process, methods to measure adherence, the scope of the CGs, and population characteristics.

**Results:**

Out of 8137 references, we included 41 primary studies conducted in eight European countries. Most followed a retrospective cohort design (19/41; 46%) and were at low or moderate risk of bias. Adherence for overall breast cancer care process (from diagnosis to follow-up) ranged from 54 to 69%; for overall treatment process [including surgery, chemotherapy (CT), endocrine therapy (ET), and radiotherapy (RT)] the median adherence was 57.5% (interquartile range (IQR) 38.8–67.3%), while for systemic therapy (CT and ET) it was 76% (IQR 68–77%). The median adherence for the processes assessed individually was higher, ranging from 74% (IQR 10–80%), for the follow-up, to 90% (IQR 87–92.5%) for ET. Internal factors that potentially impact on healthcare providers’ adherence were their perceptions, preferences, lack of knowledge, or intentional decisions.

**Conclusions:**

A substantial proportion of breast cancer patients are not receiving CGs-recommended care. Healthcare providers’ adherence to breast cancer CGs in Europe has room for improvement in almost all care processes. CGs development and implementation processes should address the main factors that influence healthcare providers' adherence, especially patient-related ones.

**Registration:**

PROSPERO (CRD42018092884).

**Electronic supplementary material:**

The online version of this article (10.1007/s10549-020-05657-8) contains supplementary material, which is available to authorised users.

## Background

Clinical guidelines (CGs) are defined as “systematically developed statements to assist healthcare providers and patients’ decisions about appropriate health care for specific clinical circumstances” [1]. These recommendations are intended to optimise patient care, reduce inappropriate practice variation, enhance the transition of research into practice, and improve healthcare quality and safety [2]. Despite the availability of CGs with different presentation formats, a constant production or updating process, their uptake, adherence, or compliance by healthcare providers is variable [3], and sometimes reported as suboptimal [4–6]. For example, it has been estimated that only 50% of patients in the United States receive CGs-compliant healthcare [4].

Shared decision-making may be influenced by the complexity of cancer care (e.g. tumour related features), patient’s characteristics, and limitations in the evidence base [5]. Barriers to healthcare providers’ adherence to CGs could be personal barriers, as the healthcare provider’s knowledge (lack of awareness or familiarity with CGs) and the provider’s attitude towards change in practice, and external barriers (the type of guideline, patient, or environment) [6]. A better understanding of barriers to CGs adherence might help healthcare providers comply with CGs recommendations, thereby improving the quality and cost-effectiveness of health care [7].

In 2018, over 400,000 incident breast cancer cases were estimated in Europe [8]. Because of the high burden of the disease and the enormous health-economic impact [9], several breast cancer CGs in Europe have been developed, reflecting decades of intensive research [2]. However, their methodological quality, evaluated with the AGREE II instrument [10], reported low scores for the “rigour of development” and “applicability” domains. The latter includes guideline implementation and resource implications [11]. Despite adherence to breast cancer CGs in Europe being associated with better survival outcomes [12, 13], healthcare providers’ adherence in usual care has not been systematically explored yet. The objective of this systematic literature review is twofold: (i) to evaluate the extent of healthcare providers’ adherence to breast cancer CGs in Europe, and (ii) to identify the barriers to CGs adherence from their perspective.

## Methods

We conducted a systematic review [14] and used the PRISMA guidance for its reporting [15]. PRISMA checklist is provided in the Additional file 1. We registered the research protocol in PROSPERO (CRD42018092884).

### Information sources and search strategy

We designed the search strategy and conducted the electronic searches, for both systematic reviews and primary studies, in MEDLINE (accessed through PubMed) and Embase (accessed through Ovid), from inception to May 2019. The strategy searched for terms related to adherence, clinical guidelines, and breast cancer. Search strategies are outlined in Additional file 2. Reference sections from retrieved systematic reviews were used to identify additional primary studies.

### Eligibility criteria and selection of studies

We sought for primary studies conducted in European countries. The addressed population was healthcare providers of breast cancer care (including primary care, oncology, radiotherapy, or others). Selected studies should measure the adherence of healthcare providers’ indications to breast cancer CGs, with any method (quantitative or qualitative) or source of data (self-reported, medical records’ assessment, interviews) or explore the barriers to adherence from healthcare providers’ perspective.

The extension of assessment could be only one recommendation or a complete guideline. We sought for studies reflecting usual care conditions. We excluded studies assessing patients’ adherence to treatment or indications, clinical trials, or studies to implement programmes to improve CGs adherence. One author (IR) screened titles and abstracts to select potentially relevant references to be evaluated on full text. Then, two authors (AVM, ENDG) independently assessed whether these studies met the eligibility criteria. Discrepancies were resolved through discussion. We managed references with Endnote version X7 software (Thomson Reuters, New York, USA).

### Data extraction and risk of bias assessment

We used tabular formats to extract main study characteristics (e.g. country, publication year, objective, year of study data, name of the guideline, guideline scope, adherence definition, number of patients, and patients’ characteristics). Primary outcomes were the proportion of patients receiving breast cancer adherent care according to CGs recommendations, by treatment modality, and the factors that impact on provider’s adherence.

We applied risk of bias assessment tools based on the study design: the AXIS tool for cross-sectional studies [16], the Quality Assessment Tool for Before-After (Pre-Post) Studies with No Control Group for non-controlled before-after studies [17], the Newcastle–Ottawa Quality Assessment Scale for prospective and retrospective cohort studies [18], and the Critical Appraisal Skills Programme (CASP) checklist for the qualitative study [19]. We followed each tools’ recommendations for the risk of bias assessment. The AXIS tool [16] assigns 0 to 10 scores and has three categories: low (1–4), moderate (5–7), and high (8–10). The Quality Assessment Tool for Before-After (Pre-Post) Studies With No Control Group [17] includes 12 criteria, and three final categories: good, fair, or poor. The Newcastle–Ottawa Quality Assessment Scale [18] uses scores from 0 (the highest risk of bias) to 9 (the lowest risk of bias). And, the Critical Appraisal Skills Programme (CASP) [19] includes ten domains, with three possible answers: “yes”, “can't tell”, and “no”.

Six authors (AVM, IR, ENDG, JP; MR, YS) were involved in the data extraction and risk of bias assessment. One author extracted data, and then two authors (YS, ENDG) contrasted the accuracy of data against full texts, cross-checked the risk of bias assessment, and completed missing information. Disagreements were solved by discussion or with the help of a third author (IR).

### Data synthesis and analysis

We considered inappropriate pooling quantitative findings due to the high variability in adherence definitions and healthcare processes assessed. Instead, we summarised results in a narrative synthesis, for which we defined and categorised breast cancer care as follows:

- The overall breast cancer care: the whole healthcare process from diagnosis to follow-up
- The overall treatment process: primary and adjuvant therapy (if applicable) including surgery, CT, ET, and RT. Follow-up procedures not included.
- Systemic therapy: comprising both CT and ET
- Pre-treatment procedures/diagnosis: staging and HER2 status assessment before the treatment process
- Surgical procedures: breast-conserving surgery (BCS), mastectomy (MA), sentinel lymph node biopsy (SLNB), axillary lymphonodectomy (ALND), or other surgery recommendations, either assessed separately or as a group.
- Therapies (assessed independently): CT and associated therapies (e.g. antiemetics); ET; Targeted anti-HER2 therapy; or RT
- Supportive measures during therapies: defined as any therapy aimed to prevent side effects for specific treatments
- Follow-up/monitoring: consultations or procedures after receiving treatment with monitoring purposes
- Primary prevention strategies: interventions to promote early diagnosis of breast cancer in asymptomatic patients.

We grouped findings based on similarities of treatment modality. To summarise the proportion of adherence, we calculated descriptive statistics (the median, Interquartile range (IQR) (including the median), and range). Data analysis included estimating if possible the overall adherence when reported disaggregated (e.g. as subgroups); selecting the least restrictive definition when several definitions were reported; and selecting the most recent publication or the one with the longest period for studies with more than one publication with a similar objective. To explore barriers to adherence, we summarised the main findings from quantitative studies reporting factors that were significantly associated with non-adherence, and narratively integrated these findings with the qualitative findings. We classified them as internal or external (related to healthcare providers or not). We explored sources of heterogeneity by the source of data (i.e. self-reported or medical records based), treatment modality, and subtype of tumour. We used Microsoft Excel for data analysis. For data reporting, we present tabular summaries and graphical representations.

## Results

### Study selection

**Fig. 1:**
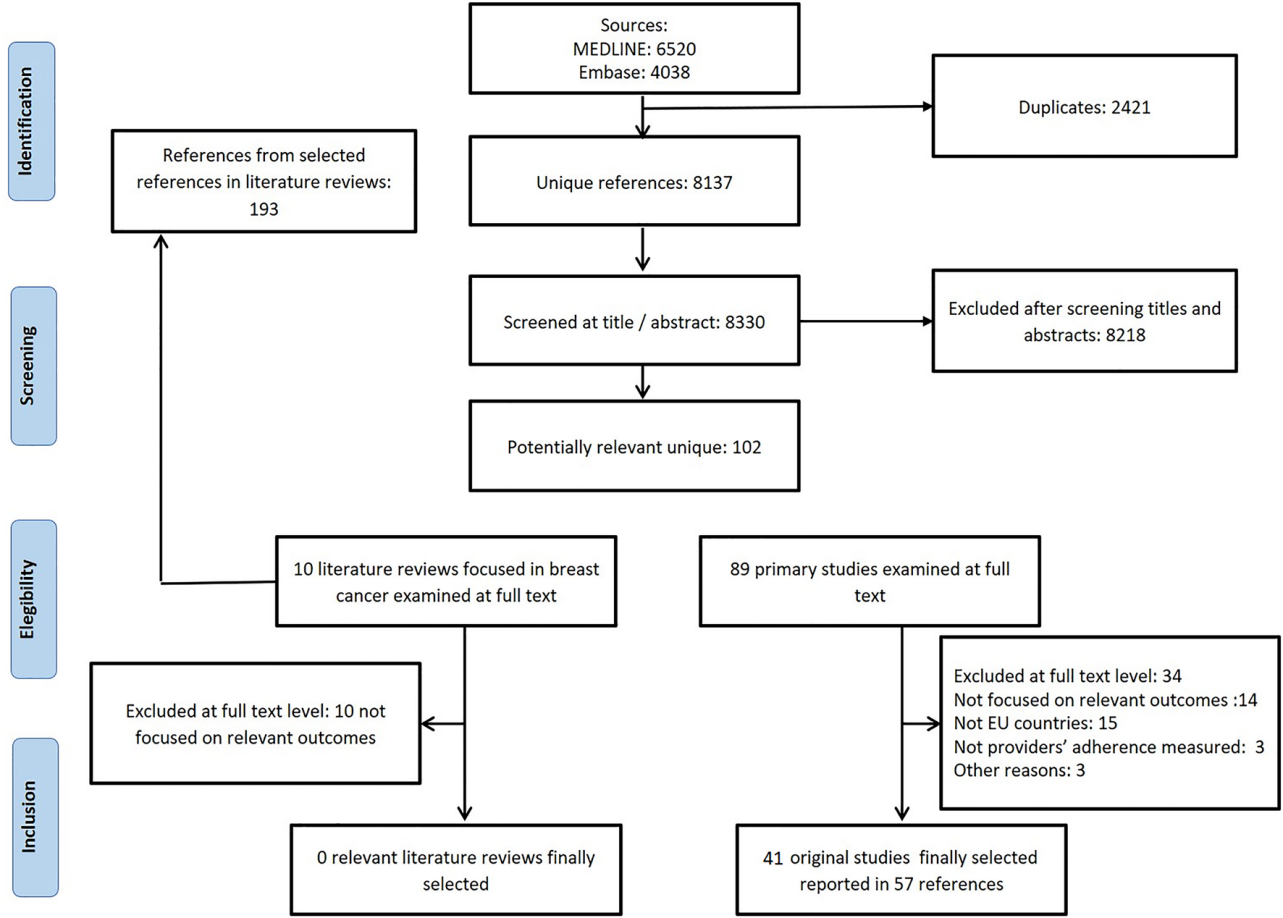
Flow chart of the screening process to identify relevant studies

Our search yielded 10,558 references. After removing duplicates, 8137 references were screened based on titles and abstracts. Of these, 102 references were selected for full-text appraisal. We finally included 57 references representing 41 primary studies. Three studies were reported in more than one publication: BRENDA I [12, 13, 20–28], BRENDA II [29–31], and OncoDoc2 [32–34]. (Fig. 1). Excluded studies alongside their exclusion rationale are available in Additional file 3.

### Study characteristics

Included studies were published between 1997 [35] and 2019 [36, 37]. These were conducted in eight European countries, with two countries leading in frequency: The Netherlands (*n* = 13, 31.7%), and Italy (*n* = 8, 19.5%). Most followed a retrospective cohort design (*n* = 19; 46.3%), the remaining included cross-sectional studies (*n* = 13; 31.7%); non-controlled before-after studies (*n* = 5; 12.2%); prospective cohorts (*n* = 4; 4.9%); a case study (*n* = 1; 2.4%), and a qualitative study (*n* = 1; 2.4%).

Two cross-sectional studies were based on a national audit [38, 39]. Still, they reported findings for two different subpopulations. Similarly, eleven retrospective studies used data from the National Cancer Registry of the Netherlands but analysed different regions or periods [40–50].

Most studies (*n* = 36, 87.8%) assessed adherence based on medical records (from hospital databases, specific registers, or national cancer registries), and their sample sizes ranged from 131 [51] to 104,201 records [44]. The remaining five studies (12.2%) were based on self-reported data, through surveys or interviews to healthcare providers [52–56], and their samples sizes ranged from 10 [56] to 202 participants [53]. Adherence to CGs recommendations was the primary outcome in 38 studies (92.6%), while three focused on factors that influence adherence [32, 47, 56]. Sixteen studies (39.0%) measured adherence to CGs for more than one process of care (Table 1, Additional file 4).

**Table 1:** Characteristics of selected studies (*n* = 41)

Characteristics	*n*	%	References
Country			
The Netherlands	13	31.7	[40–50, 60, 65]
Italy	8	19.5	[38, 39, 52, 55, 57, 58, 61, 66]
Germany^1^	6	14.6	[12, 13, 20–31, 36, 62, 64, 67, 68]
France^1^	6	14.6	[33–35, 51, 63, 69, 70]
UK	4	9.8	[53, 54, 56, 71]
Denmark	2	4.9	[72, 73]
Croatia	1	2.4	[37]
Belgium	1	2.4	[74]
Study design			
Retrospective cohort^1^	19	46.3	[13, 25–27, 36, 40, 41, 44–51, 57, 62, 66, 71, 73]
Cross-sectional	13	31.7	[37–39, 42, 52–55, 58, 63–65, 70]
Non-controlled before-after	5	12.2	[35, 59–61, 69]
Prospective cohort^1^	2	4.9	[29–31, 74]
Case study^1^	1	2.4	[32–34]
Other (qualitative)	1	2.4	[56]
Publication year^a^			
1999–2008	12	21.1	[35, 38, 39, 41, 45, 46, 52, 53, 60, 69, 71]
2009–2011	13	22.8	[13, 21, 24, 25, 32, 40, 50, 51, 58, 63–66]
2012–2014	11	19.3	[12, 22, 26, 33, 34, 42, 48, 55, 61, 68, 70]
2015–2017	16	28.1	[20, 23, 27, 28, 30, 31, 43, 44, 47, 49, 56, 59, 62, 67, 72, 74]
2018–2019	5	8.8	[29, 36, 37, 54, 73]
Guideline scope^b^			
Treatment^1^	31	75.6	[12, 13, 20–30, 32, 35, 36, 40, 41, 43–45, 47, 48, 50, 51, 57–64, 66, 68, 69, 71–74]
Follow-up	4	9.8	[35, 42, 55, 57, 58, 69]
Preventive measures	4	9.8	[37, 49, 53, 56, 70, 74]
Diagnosis	4	9.8	[35, 58, 61, 65]
Risk of bias^c^			
Low	25	62.5	[13, 30, 35, 36, 40–44, 46, 48, 49, 55–58, 61, 65, 66, 70–72, 74, 75]
Moderate	14	35.0	[37–39, 45, 47, 50–54, 59, 60, 63, 69]
High	1	2.5	[64]

^1^Reported in more than one separate publications

^a^Percentages calculated over the total number of publications (*n* = 57)

^b^The total exceeds 100% since some guidelines comprised more than one scope

^c^Percentages over 40 studies, a case study was not evaluated

Thirty-one studies (75.6%) reported how researchers measured the adherence to CGs. Most of these studies (22/31, 71%) assessed adherence contrasting healthcare providers’ indications for breast cancer patients against a selection of CGs recommendations. Six studies (6/31, 19.4%) addressed adherence as a process indicator integrated in the quality assurance programme of their institution [57–62]. In three studies (3/31, 9.7%), a “tumour board”, or a multidisciplinary team of physicians, was involved in the clinical pathway of treatment decision [29, 31, 32, 63]. The definitions of adherence were variable across studies. They included one or more of the following components: non-adherence definition, classification for non-adherent treatment, threshold criterion, reference to the currency of guideline, or to the relevance of patients’ profile (Additional file 5).

### Risk of bias

We assessed the risk of bias for 40 studies; the case study [32–34] was not considered as suitable for this assessment. Most were at low (25/40, 62.5%) or moderate risk of bias (14/40, 35.0%). Only one study (1/40, 6%) was considered to be at high risk of bias, mainly due to concerns regarding selection bias in data analyses [64] (Table 1, Additional file 6).

### Adherence to breast cancer CGs recommendations for healthcare processes

**Fig. 2:**
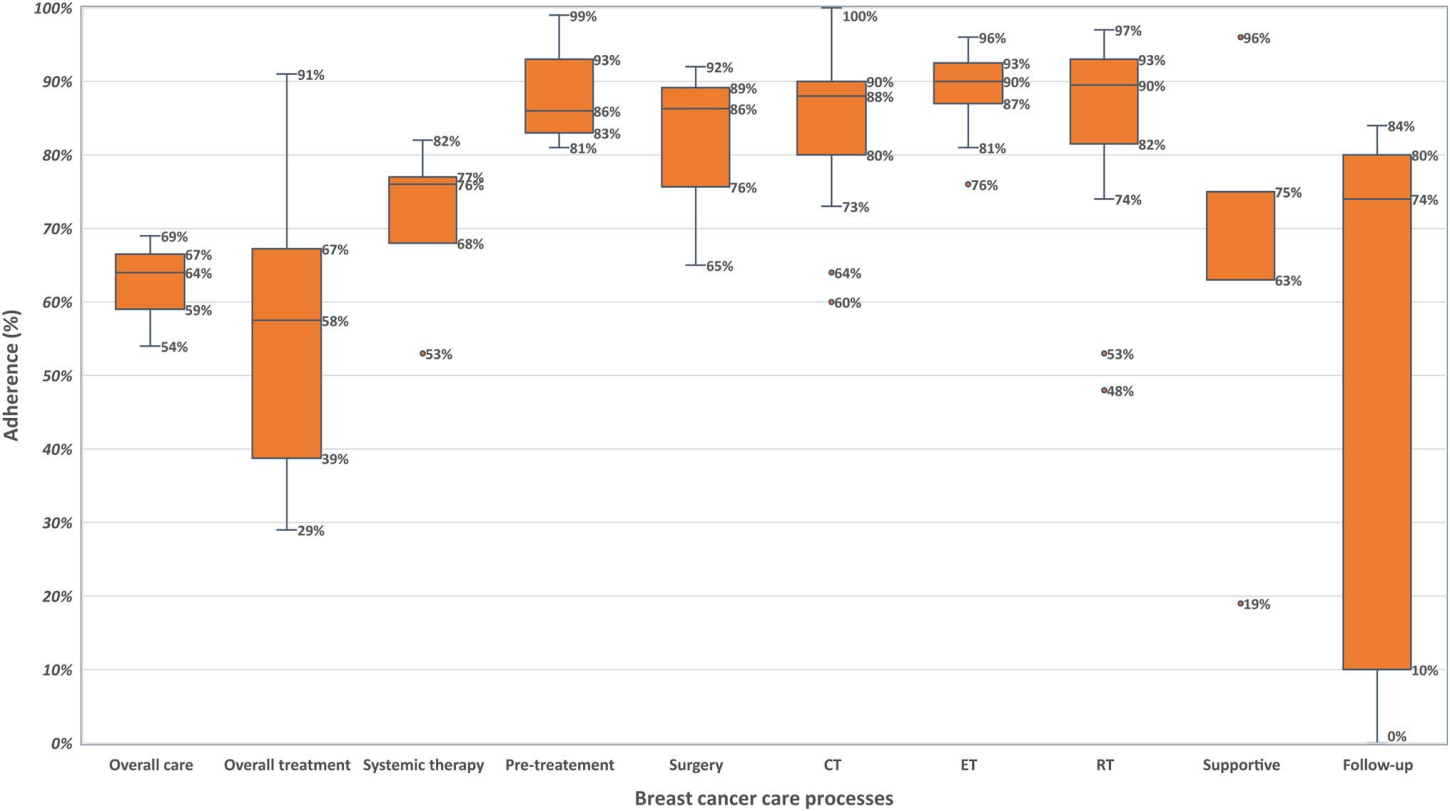
Median adherence proportions for overall breast cancer care and individual therapies. The square inner line represents the median, while the upper and lower borders, the interquartile ranges. The bars represent the “minimum” and “maximum” values. Outliers are shown as circles. *CT* chemotherapy, *ET* endocrine therapy, *RT* radiotherapy

Main findings reported by treatment modality, and clinical guidelines are available in Table 2 and Fig. 2.

**Table 2:** Adherence levels by treatment modality

Study^1^	Guideline	Overall care* or overall treatment	ST	Pre-treatment/diagnosis	Surgical procedures	CT	ET	RT	Anti HER2	Supportive measures	Follow -up
Andreano 2017 [57]	ESMO 2015	69%*	76%	81%	97%	–	–	RT after BCS 84%	–	–	84%
Balasubramanian 2003 [71]	North Trent breast cancer group guidelines for treatment and referral (1998)		82%	–	–	90%	–	74%	–	–	–
Barni 2011 [58]	Italian Association of Medical Oncology (AIOM)	64%*–71%		93%	BCS 74%; ALND 68%; SLNB 57%	100%	90%	92%		–	0%
Boskovic 2017 [74]	Bone health guidelines		–	–	–	–	–	–	–	32–75%	–
BRENDA I Ebner 2015 [67]	German National Step 3 (S3) (younger vs older)		67% vs 47%	–	86.3% vs 88.3%	88% vs 68%	91% vs 87%	95% vs 83%	–	–	–
BRENDA I Ebner 2015a [20]	German National Step 3	61%	–	–	91%	74%	75%	83%	–	–	–
BRENDA I Hancke 2010 [21]	German National Step 3		–	–	BCS: 87%; MT: 52%	Recommended: 56%; not recommended: 95%	Recommended: 88%; not recommended: 92%	Recommended: 86%; not recommended: 88%	–	–	–
BRENDA I Schwentner 2012a [12]	German National Step 3	33% vs 57% overall: 54%	–	–	BCS: 86%	74%	–	84%	–	–	–
BRENDA I Schwentner 2012b [22]	German National Step 3	BBC 15%	–	–	–	–	–	–	–	–	–
BRENDA I Schwentner 2013 [68]	German National Step 3	–	TNBC: 44%; non-TNBC: 70%	–	TNBC 86%; non-TNBC 87%	TNBC 53%; non-TNBC 87%	–	TNBC: 90%; non-TNBC 92%	–	–	–
BRENDA I Van Ewijk 2015 [23]	German National Step 3	–	59%	–	87.6%	78%	91%	91%	–	–	–
BRENDA I Varga 2010 [24]	German National Step 3	–	54%	–	87%	80%	81%	87%	–	–	–
BRENDA I Wöckel 2010 [25]	German National Step 3 2004	52%	–	–	BCS: 85% MT: 85%	71%	85%	84%	–	–	–
BRENDA I Wöckel 2014 [26]	German National Step 3	–	–	–	–	–	–	87%	–	–	–
BRENDA I Wökel 2010a [13]	German National Step 3	48%	–	–	–	–	–	–	–	–	–
BRENDA I Wollschlager 2017 [27]	German National Step 3	–	Charlson 0: 68% Charlson 1–2: 66% Charlson ≥ 3: 58%	–	–	–	–	–	–	–	–
BRENDA I Wolters 2015 [28]	German National Step 3	53%	–	–	–	–	–	–	–		–
BRENDA II Stuber 2017 [31]	German National Step 3	–	–	–	–	–	92%	–	–	–	–
BRENDA II Leinert 2019 [29]	German National Step 3	–	–	–	–	High risk: 90%; low risk: 100%	–	–	–	–	–
BRENDA II Schwentner 2016 [30]	German National Step 3	–	–	–	–	85%		–	–	–	–
Bucchi 2009 [66]	1995, 1998, 2001 Gallen International Conferences guidelines		53%	–	–	–	–	–	–	–	–
de Munck 2011 [40]	NABON (National Breast Cancer Organization of The Netherlands)		–	–	–	–	–	–	94%	–	–
de Roos 2005 [41]	CCN guidelines	69%	–	–	–	–	–	–	–	–	–
DURTO 2003 [38]	St Gallen International Consensus Panel		–	–	–			–	–	96%	–
Heins 2017 [43]	Dutch National Cancer Treatment guidelines for breast cancer	–	–	–	–	90–99%	90–95%	–	–	–	–
Holm-Rasmussen 2017 [72]	Danish Breast Cancer Group guideline	–	–	–	SLNB 76%	–	–	–	–	–	–
Jacke 2015 [59]	S1-guidelines (1996–1997) vs S3-guidelines (2003–2004)	15% vs 34%	–	–	ALND 81% vs 81%	75% vs 93%	70% vs 84%	BCS + RT: 29% vs 53%	–	–	–
Jensen 2018 [73]	Danish Breast Cancer Group guidelines	–	–	99%	95%	97%	96%	80–97%	–	–	–
Kuijer 2017 [44]	Dutch AST guidelines: 2005–2008, 2008–2012 and 2012–2014	–	–	–	–	78%	88%	–	–	–	–
Lebeau 2011 [63]	French National CPGs	12–29%	–	–	65%	73%	88%	48%	–	–	–
Liebrich 2011 [64]	2006 AGO-S3 German Cancer Society	–	–	–	–	–	–	–	77%	–	–
Mille 2000 [69]	Centre Regional Leon Bernard (CRLB) guideline (1993 vs 1995)	–	–	–	–	–	–	–	–	–	First–year 16% vs 74%
Mylvaganam 2018 [54]	British best practice guidelines for implant-based breast reconstruction 2015	–	–	–	17–50%	–	–	–	–	19–100%	–
Natoli 2014 [55]	National and International Oncology societies	–	–	–	–	–	–	–	–	–	10%
OncoDoc2 Bouaud 2011 [32] Seroussi 2012, 2013 [33, 34]	Cancer Est CPGs	91%	–	–	–	–	–	–	–	–	–
Ottevanger 2004 [60]	CCCE National guideline (1988 vs 1996)	–	–	–	BCS 39% vs 35%; MT: 56% vs 54%	91% vs 88%	–	75% vs 93%	–	–	–
Poncet 2009 [51]	French National guidelines and regional CGs	–	–	–	–	–	–	–	National 31%–Regional 83%	–	–
Ray-Coquard 1997 [35]	Guideline in 1993 (1993 vs 1995)	19% vs 54%*	–	75% vs 86%	96% vs 92%	71% vs 85%	72% vs 94%	83% vs 93%	–	–	31% vs 80%
Ray-Coquard 2012 [70]	French and international guidelines to manage CT-induced anaemia	–	–	–	–	95%	–	–	–	–	–
Roila 2003 [39]	St Gallen International Consensus Panel	–	–	–	–	60%	81%	–	–	–	–
Sacerdote 2013 [61]	Piedmont guidelines (2002 vs 2004)	–	–	–	BCS 86% vs 93%	66% vs 64%	94% vs 94%	88% vs. 88%	–	–	–
Schaapveld 2004 [46]	Regional treatment CCCN Guidelines	–	–	–	–	89%	–	–	–	–	–
Schaapveld 2005 [45]	CCCN Guidelines	–	–	–	BCS: 95%; MRM: 90.6%	–	–	–	–	–	–
Schrodi 2015 [62]	German National Step 3 (1999 vs 2010)	–	–	–	BCS 72%; SLNB: 0.2% vs 51%	–	–	–	–	–	–
van de Water 2012 [48]	Dutch AST guidelines: 2005–2008, 2008–2012 and 2012–2014	< 65 years: 62% ≥ 75 years: 56%	–	–	–	–	–	–	–	–	–
Van Ryckeghem 2019 [37]	European Organisation for Research and Treatment of Cancer guidelines (EORTC 2010)	–	–	–	–	–	–	–	–	75%	–
Vercauteren 2010 [65]	Dutch national CGs for breast cancer screening and diagnosis, American College of Radiology CGs	–	–	Adding US: 94%; not additional: 83%	–	–	–	–	–	–	–
Visser 2016 [49]	HERA (Herceptin adjuvant study)	–	–	–	–	–	–	–	–	63%	–
Weggelaar 2011 [50]	CCN guidelines	–	77%	–	–	90%	76%	91%	–	–	–
Wimmer 2019 [36]	German National Step 3 (2017)	–	–	–	–	–	–	97%	–		–

^1^Sorted in alphabetical order

*Including follow-up

*BBC* bilateral breast cancer, *BCS* breast-conserving surgery, *CT* chemotherapy, *ET* endocrine therapy, *HER2* human epidermal growth receptor, *MRM* modified radical mastectomy, *MT* mastectomy, *SLNB* sentinel lymph node biopsy, *TNBC* triple-negative breast cancer, *US* ultrasonography. Preventive measures procedures described in the text

#### Overall breast cancer care

Adherence to CGs for the overall breast cancer care was measured only in three studies with a range from 54 and 69% [35, 57, 58] and included patients receiving treatment from 1995 to 2012. These studies varied in what process they considered as part of overall care: one included RT, CT, ET, initial examination, and follow-up indications and found that only half of the clinicians were adherent to CGs (54%) [35]; the second study evaluated nine quality indicators for diagnosis, surgery, therapy, and follow-up, and found 64% of adherence to CGs [58]; and the third measured seven process indicators of breast cancer care including follow-up and found 69% of adherence with the 80% of cut-off, and 38% when it increased to 90% [57].

#### Overall treatment process

Six studies addressed the overall treatment process (surgery, CT, ET, and RT). These studies represented patients receiving treatment in the period from 1991 to 2009 [28, 32, 41, 48, 59, 63]. The median adherence was 57.5% (IQR 38.8–67.3%), and ranged from 29 [63] to 91% [32]. A subgroup analysis of the BRENDA I study [22] found that only 15% of patients with bilateral breast cancer (BBC) received a compliant treatment, requiring 100% of compliance to define adherence.

#### Systemic therapy

Five studies addressed systemic therapy (CT and ET indications). These studies included patients receiving treatment in the period from 1992 to 2012 [27, 50, 57, 66, 71]. The median adherence for systemic therapy was 76% (IQR 68–77%), and ranged from 53 [66] to 82% [71].

### Adherence to breast cancer CGs—procedures or therapies (assessed separately)

#### Pre-treatment procedures

Five studies addressed the procedures before starting treatment. [35, 57, 58, 65, 73]. These procedures were initial examination [35], indicating mammography before surgery [57, 58]; using ultrasonography after mammography when applicable [65]; and assessing HER2 receptors status before surgery [73]. The median adherence for pre-treatment procedures was 86% (IQR 82–96%), and ranged from 81%, for indicating mammography [57], to 99%, for HER2 status assessment [73].

#### Surgical procedures

Three studies assessed compliance for more than one surgical procedure. These studies included patients receiving treatment in the period from 1992 to 2008 [20, 35, 63].The median adherence for surgical procedures was 86.3% (IQR 75.7–89.2%), and ranged from 65 [63] to 92% [35].

Moreover, eleven studies measured adherence for individual surgical procedures which included (1) breast-conserving surgery (BCS) [45, 58, 60–62], the median adherence was 74% (IQR 75.7–93%), and ranged from 35 [60] to 95% [45]; (2) mastectomy (MA) [25, 45, 60], the adherence ranged from 54 [60] to 91% [45]; (3) SLNB [58, 62, 72], from 51 [62] to 76% [72]; (4) ALND [58, 59, 73], from 68 [58] to 81% [59]; and (5) other indicators included organisational indicators [54] or “breast surgery without needing a second surgery” [57], which reported 17% and 97% of adherent treatment, respectively.

#### Chemotherapy

Fifteen studies addressed CT, including patients receiving treatment in the period between 1992 and 2016 [20, 29, 35, 39, 43, 44, 46, 50, 58–61, 63, 71, 73]. The median adherence was 88% (IQR 80–90%), and ranged from 60 [39] to 100% [58]. Additionally, one study [70] assessed compliance with CGs recommendations for the treatment of CT-induced anaemia and found that 95% of patients received erythropoiesis-stimulating agents (ESAs) according to CGs.

#### Endocrine therapy

Twelve studies addressed recommendations for ET, including patients receiving treatment in the period between 1992 and 2016 [20, 31, 35, 39, 43, 44, 50, 58, 59, 61, 63, 73]. The median adherence was 90% (IQR 87–92.5%), and ranged from 76 [50] to 96% [73].

#### Targeted anti-HER2 therapy

Three studies assessed recommendations for the use of anti-HER2 therapy, including patients receiving treatment in the period between 2003 and 2009 [40, 51, 64]. The adherence values were 31% [51], 77% [64], and 94% [40].

#### Radiotherapy

Twelve studies addressed recommendations for RT, including patients receiving treatment in the period between 1992 and 2016 [26, 35, 36, 50, 57–61, 63, 71, 75]. The median adherence was 89.5% (IQR 81.5–93%), and ranged from 48 [34] to 97% [36, 73].

#### Supportive measures during therapies

Five studies [37, 38, 49, 54, 74] addressed procedures aimed to prevent adverse events during treatment, including patients receiving treatment in the period between 1996 and 2017. The median adherence was 75% (IQR 63–75%, and ranged from 19 [46] to 96% [37]. These recommendations included (1) using antiemetics to avoid acute or delayed emesis induced by chemotherapy [37]; (2) applying measures to prevent surgical infections [46]; (3) using primary prophylaxis with granulocyte colony-stimulating factors in patients receiving chemotherapy [27]; (4) monitoring left ventricular ejection fraction (LVEF) during anti-HER2 therapy [76], and (5) indicating vitamin D and calcium in patients receiving adjuvant non-steroidal aromatase inhibitors [75].

#### Follow-up

Six studies addressed follow-up indications, including patients receiving treatment in the period between 1995 and 2013 [35, 42, 55, 57, 58, 69]. The median adherence was 74% (IQR 10–80%), and ranged from 0 [58] to 84% [57]. One study reported that “consultations and mammograms for follow-up purposes were excessive”, and this was the reason for non-compliance [42].

#### Primary prevention strategies

One study [45] addressed primary prevention strategies in primary care, related to the management of women with concerns about familial breast cancer, and found that 80% of the general practitioners were compliant with CGs.

### Barriers for healthcare providers’ adherence to breast cancer CGs

**Fig. 3:**
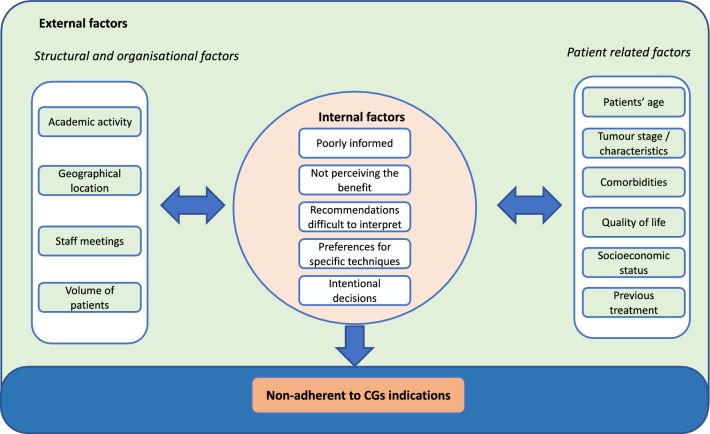
Barriers for healthcare providers’ adherence to breast cancer CGs. Based on main categories proposed by Cabana et al. [6]

Sixteen studies described and analysed potential barriers for adherence to CGs, and factors associated with non-adherent indications. These barriers were categorised as internal and external factors [6]; the latter included patient-related factors and structural/organisational barriers (Table 3, Fig. 3).

**Table 3:** Barriers that impact on healthcare providers’ adherence to breast cancer CGs

Factor	Main findings
Internal factor	
Practitioners' perceptions and preferences	∙ Clinicians were poorly informed about preventive therapy or perceived lack of benefit of preventive therapy and experienced difficulties interpreting guidelines [56] ∙ The considerable variation in BCS rates is more consistent with variations in surgeon preferences than the patient’s choice [45] ∙ Intentional and conscious healthcare providers’ decisions [34]
External factor—patient-related factors	
Patient’s age	∙ Treatment adherence was significantly lower for surgery, RT, and systemic therapy in women aged 80 years and older, and all modalities were applied much less frequently, except for endocrine therapy which was more frequently applied in the oldest [50] ∙ Non-compliance with clinical decisions for treatment was associated with older patient age [63] ∙ Deviations from the initial therapy decision were more frequent in older patients (≥ 75 years) than in younger ones [30] ∙ Non-compliance has concerned elderly patients age > 80 years in pre-surgery decisions [33, 34] ∙ Adherence to treatment guidelines was affected by age at diagnosis [48] ∙ The effect of non-adherence was stronger in the oldest [20] ∙ Non-adherence on RT and CT increased with age [21] ∙ One reason to withhold trastuzumab was older age [40, 64] ∙ Adherence was markedly lower for elderly patients; either ALND or RT was omitted [45] ∙ Guideline adherence was significantly lower in TNBC, most pronounced in the > 65 years subgroup [68] ∙ Tumour board decision against ET was associated with the younger age of patients [31] ∙ The use of SLNB was significantly higher in younger patients (< 40 years) [72]
Comorbidities	∙ Non-guideline-adherent treatment was associated with comorbidities [20], Charlson index [27] “particular cases” [32] ∙ Most common reasons to withhold trastuzumab were cardiovascular disease [40] ∙ Variation was in part but not entirely attributable to comorbidities [64] ∙ Tumour board recommendation against CT was significantly more frequent in patients with cognitive impairment [29]
Tumour stage/tumour characteristics	∙ Non-guideline-adherent treatment was associated with higher tumour stages [20] ∙ Non-compliance on RT was associated with lymph node involvement or peritumoral vascular invasion. Within the overall treatment sequence, it was associated with positive lymph nodes, and grade III versus grade I [63] ∙ Non-compliance was associated with a micro-invasive tumour in re-excision decisions, and with HR + and Her2+ in adjuvant decisions [33, 34] ∙ Compliance with SLNB with BCS was significantly higher in patients with tumour size ≥ 50 mm, Van Nuys classification group III, palpable lesion, and upper lateral quadrant of the breast location [72]
Gene expression profile	∙ The use of gene expression profiles (GEP) was independently associated with an increased risk of receiving CT in clinical low-risk patients and with a lower risk of CT administration in high-risk patients [47]. Adherence to the GEP result was higher in high-risk patients with a discordant GEP result compared to low-risk patients with a discordant GEP result [47]
Quality of life (QoL)	∙ Tumour board decision against ET was associated with reduced QoL [31]. If the QoL was good, higher age was not related to deviation [30]
Previous treatment	∙ Women who received RT had excessive follow-up consultations compared to did not receive it [42] ∙ Non-adherence was associated with the absence of prior axillary surgery in adjuvant decisions [33] ∙ In high-risk febrile neutropenia category, adherence to primary prophylaxis with granulocyte colony-stimulating factors (PPG) was more common among patients receiving dose-dense therapy than those receiving the classical chemotherapy [37]
Socioeconomic status	∙ Low socioeconomic status (SES) patients were more likely to be undertreated with CT than high SES patients, however no association with ET. For ethnicity, no association with CT or ET was observed [44]
External factor—structural and Organisation factors	
Geographic region and academic status	∙ Non-compliant decisions were mainly “choices of multidisciplinary staff meetings” [34] ∙ More adherence in research centres, in Northern Italy [38], and in one region of care versus another [63] ∙ The use of SLNB in patients who underwent BCS was significantly higher in low-volume departments. While for women who underwent a mastectomy, SLNB was higher in high-volume departments [72]

*BCS* breast-conserving surgery, *RT* radiotherapy, *CT* chemotherapy, *ET* endocrine therapy, *SLNB* sentinel lymph node biopsy, *ALND* axillary linfatic node dissection, *TNBC* triple-negative breast cancer, *GEP* gene expression profile, *QoL* quality of life, *SES* socioeconomic status

Internal factors, represented by healthcare provider-related factors for non-adherence to CGs, include their perceptions, preferences, knowledge, and attitudes regarding CGs recommendations. A qualitative study [56] explored the barriers for the implementation of CGs for preventive treatment for women at increased risk of breast cancer. According to providers’ perceptions, the reasons for non-adherence were the perceived lack of benefit of the interventions, being poorly informed and finding difficulties interpreting recommendations [56]. Another retrospective study reported that non-adherent indications in surgery reflected providers’ preferences for using specific techniques [45].

In other cases, these non-adherent indications would be intentional and conscious healthcare providers' decisions, as described in a case study [34] conducted within an [“optimal”] setting, where a CGs-based clinical decision support system (OncoDoc2) was routinely applied. They found that all non-compliant decisions concerned mostly a group of patients and decisions (i.e. elderly patients in pre-surgery decisions, patients with micro-invasive tumour in re-excision decisions, and patients with positive hormone receptors and HER2+ in adjuvant decisions). These discordances were found mainly in areas where scientific evidence is lacking, and the non-adherence behaviour, in this case, was actually intentional and conscious [34] (Table 3, Fig. 3).

External factors include the patient-related factors and structural factors. The patients’ age was the patient-related factor that appeared consistently associated with non-adherent treatment [20, 21, 30, 31, 33, 34, 40, 45, 48, 50, 63, 64, 68, 72]. In comparison with younger patients, older women were less likely to receive CGs concordant surgery, CT and RT, but were more likely to receive guideline-concordant ET. The intrinsic tumour characteristics were also associated with non-adherent behaviours, such as the gene expression profile [47] having triple-negative breast cancer [12, 68] or being diagnosed with higher tumour stages [20, 63]. Non-adherence was also associated with comorbidities [20, 27, 29, 32, 40, 64] [e.g. in patients with cardiovascular diseases, it was more frequent to withhold trastuzumab [40], or in patients with cognitive impairment it was more frequent the delay starting chemotherapy [29]]. Other patient-related factors included the quality of life (QoL) and low socioeconomic status (SES) [31]. Poor QoL was associated with non-adherence to ET [31]. If the QoL was good, older age was not related to deviation [30]. Low SES patients were more likely to be undertreated with CT than patients with higher SES; however, an association with ET was not found [29, 44]. Previous treatment also influenced non-adherence, e.g. RT was associated with an increased follow-up consultation [42], the absence of prior axillary surgery, with non-adherence to adjuvant decisions [33], and the type of CT received [37] with non-indicating therapies to prevent side effects.

The structural factors represent the environmental or organisational characteristics of the healthcare system were associated with non-adherent behaviour. One study reported that non-compliant decisions were mainly “choices of multidisciplinary staff meetings” [34]. Other factors associated with adherence to CGs recommendations were the academic activity in the organisation [38], geographical location [63], and the volume of departments [72] (Table 3, Fig. 3).

## Discussion

### Main findings

We synthesised the literature exploring the healthcare providers’ adherence to breast cancer CGs published in the last 20 years across eight European countries. Most followed a retrospective cohort design and were considered to have a low or moderate risk of bias. Adherence for overall breast cancer care process (from diagnosis to follow-up) ranged from 54 to 69%, for the overall treatment process (including surgery, CT, ET, and RT) the median adherence was 57.5% (IQR 38.8–67.3%), while for systemic therapy it was 76% (IQR 68–77%). The median adherence values for the processes assessed individually were higher, ranging from 74% (IQR 10–80%) for the follow-up to 90% (IQR 87–92.5%) for ET. Factors that potentially impact on healthcare providers’ adherence were internal: their perceptions, preferences, lack of knowledge, or intentional decisions; and external: the patient-related and structural factors. The most consistent factor for non-adherence was the age of patients.

### Our results in the context of previous research

Previous systematic reviews addressing adherence of healthcare providers to CGs recommendations but out of the scope of breast cancer care [76–79], found similar results to our review, these studies reported a wide range of adherence values, depending on the specific criteria evaluated or the treatment modality. From 8.2 to 65.3%, for a set of CGs [76]; from 5 to 95% for different recommendations in the management of acute coronary syndromes [77]; and from 0.3 to 100% in antibiotic prophylaxis [78]. One study found 50% of adherence for cancer-associated thrombosis [79], lower than the median estimated for the overall treatment process in breast cancer care (58%).

Previous systematic reviews have also explored the main barriers to healthcare providers’ adherence in other healthcare areas [77, 78, 81, 82]. The factors they reported are consistent with our findings: the internal factors related to health professionals' limited skills or competences to use a therapy, and their poor knowledge, a low level of awareness, familiarity or confidence with the commonly used therapy (prior to CGs-recommended therapy), or a perceived lack of benefit of the "new" therapy [80–82]. They also pointed out as internal factors those related to intentional non-adherence [77]. Those highlighted as external factors include patient-related factors, such as comorbidities and patient-level barriers [78, 80], as well as structural factors, such as organisational characteristics like being a teaching hospital [78].

Other additional factors identified in previous studies included poor organisational- or institutional-level support, inadequate peer support among health professionals, the complex nature of some therapies or guidelines [80], the expectation that compliance is mandatory [4], or the patients' preferences or demands [4, 81].

### Strengths and limitations

Our study has several strengths. The broad eligibility criteria we applied helped to capture the complete picture of the healthcare providers’ adherence to breast cancer CGs in Europe. We included studies with any design reporting the measure of adherence even for single CGs recommendations. The detailed data extraction process helped us to provide a synthesis of adherence proportions by treatment modality, although definitions or selected recommendations were variable. We could identify different reporting elements across definitions and methods to measure adherence. For factors associated with healthcare providers’ adherence, we adapted a conventional classification, which highlights factors to be considered by breast cancer CGs developers and other relevant stakeholders.

Our study also has some limitations. Included studies were conducted in eight European countries, with more than half of them coming from two countries (The Netherlands and Italy). Therefore, we should be cautious when interpreting results and cannot extrapolate our findings to other European countries. Since we included studies of patient cohorts from the 1990s, when the implementation of guidelines was starting, we might be underestimating CGs adherence values. We identified only one qualitative study exploring factors for non-adherence to breast cancer CGs, possibly explained by the type of databases we selected. Another important limitation is that guideline non-adherence data did not differentiate deliberate guideline deviations from unjustified practice variation. This was not possible to explore as most of the included studies did not specify the reasons for non-adherence. Other factors, like changes between versions or discrepancies between several national and international guidelines, could have potentially influenced guideline deviations. However, this analysis was not feasible.

### Implications for practice and research

This systematic review summarises for first-time healthcare providers’ adherence to breast cancer guidelines in Europe. Even though observed median proportions seem to be acceptable for most specific treatments, there is still room for improvement for healthcare providers’ adherence, especially for supportive measures during therapy as well as during follow-up. Advances in breast cancer screening and treatment have reduced breast cancer mortality across the age spectrum in the past decade [73, 74]. However, we identified that the most consistent external factor associated with non-adherence to CGs was the older age of patients. Breast CGs might not adequately address this subpopulation, or they may represent a population where the evidence to develop breast cancer guidelines is scarce.

We found high variability in the methods used to measure, define, and report adherence. Future studies should provide the rationale to define adherence with enough transparency and should always consider the strength of a recommendation in their selection. In usual care, healthcare providers may not be aware of the standards required for CGs and may be tempted to select low-quality recommendations [74]. Hence, to facilitate the use of evidence supporting healthcare recommendations, guideline developers should use rigorously developed presentation formats (e.g. decisions aids, clinical decision trees). In agreement with other authors [75, 76], we believe it is necessary to provide a selection of relevant, reliable, and reproducible definitions for unwarranted clinical variation in healthcare [76], in this case for breast cancer care. Use of robust methods to measure adherence, avoiding the selection or performance bias, with appropriate blinding of assessment, will help to evaluate this process in a more reliable and reproducible way. The development of more qualitative research to capture breast cancer healthcare providers' perspective should be fostered. Furthermore, health care providers should register the reasons for non-adherence to facilitate that real-world data inform guideline updating.

## Conclusions

A substantial proportion of breast cancer patients appear not to be receiving CGs-recommended care. Aligning healthcare provider’s decisions with breast cancer CGs recommendations in European countries should be improved for almost all processes of care, especially for preventive therapies and follow-up. Knowing the reasons for non-compliance is essential to understand these deviations. The development and implementation of CGs for breast cancer patients should address relevant patient-related factors to enhance the applicability of CGs in clinical care**.**

## Electronic supplementary material

Below is the link to the electronic supplementary material.

Supplementary file1 (DOCX 305 kb)

## Data Availability

The datasets used or analysed during the current study are available from the corresponding author on reasonable request.

## Abbreviations

**CGs**: Clinical guidelines
**RT**: Radiotherapy
**CT**: Chemotherapy
**ET**: Endocrine therapy or hormonal therapy
**BCS**: Breast-conserving surgery
**MT**: Mastectomy
**ALN**D: Axillary lymph node dissection
**TNBC**: Triple-negative breast cancer
**SLNB**: Sentinel lymph node biopsy
**HER2**: Human epidermal growth receptor 2
