# Supplementary material for: Healthcare providers’ adherence to breast cancer guidelines in Europe: a systematic literature review
Source: Breast Cancer Res Treat. 2020 May 6;181(3):499–518. doi: 10.1007/s10549-020-05657-8 (PMC7220981; doi:10.1007/s10549-020-05657-8)
Supplement: Supplementary file 1 — Supplementary file1 (DOCX 305 kb) [file 10549_2020_5657_MOESM1_ESM.docx]

### Additional file 1 PRISMA Checklist


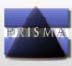
**PRISMA 2009 Checklist**

| **Section/topic** | **#** | **Checklist item** | **Reported on page #** |
| --- | --- | --- | --- |
| **TITLE** | | |  |
| Title | 1 | Identify the report as a systematic review, meta-analysis, or both. | 1 |
| **ABSTRACT** | | |  |
| Structured summary | 2 | Provide a structured summary including, as applicable: background; objectives; data sources; study eligibility criteria, participants, and interventions; study appraisal and synthesis methods; results; limitations; conclusions and implications of key findings; systematic review registration number. | 3 |
| **INTRODUCTION** | | |  |
| Rationale | 3 | Describe the rationale for the review in the context of what is already known. | 5 |
| Objectives | 4 | Provide an explicit statement of questions being addressed with reference to participants, interventions, comparisons, outcomes, and study design (PICOS). | 5-6 |
| **METHODS** | | |  |
| Protocol and registration | 5 | Indicate if a review protocol exists, if and where it can be accessed (e.g., Web address), and, if available, provide registration information including registration number. | 6 |
| Eligibility criteria | 6 | Specify study characteristics (e.g., PICOS, length of follow-up) and report characteristics (e.g., years considered, language, publication status) used as criteria for eligibility, giving rationale. | 6 |
| Information sources | 7 | Describe all information sources (e.g., databases with dates of coverage, contact with study authors to identify additional studies) in the search and date last searched. | 6 |
| Search | 8 | Present full electronic search strategy for at least one database, including any limits used, such that it could be repeated. | Additional file 1 |
| Study selection | 9 | State the process for selecting studies (i.e., screening, eligibility, included in systematic review, and, if applicable, included in the meta-analysis). | 6 |
| Data collection process | 10 | Describe method of data extraction from reports (e.g., piloted forms, independently, in duplicate) and any processes for obtaining and confirming data from investigators. | 7 |
| Data items | 11 | List and define all variables for which data were sought (e.g., PICOS, funding sources) and any assumptions and simplifications made. | 7 |
| Risk of bias in individual studies | 12 | Describe methods used for assessing risk of bias of individual studies (including specification of whether this was done at the study or outcome level), and how this information is to be used in any data synthesis. | 7 |
| Summary measures | 13 | State the principal summary measures (e.g., risk ratio, difference in means). | 8 |
| Synthesis of results | 14 | Describe the methods of handling data and combining results of studies, if done, including measures of consistency (e.g., I^2^) for each meta-analysis. | 8 |

Page 1 of 2

| **Section/topic** | **#** | **Checklist item** | **Reported on page #** |
| --- | --- | --- | --- |
| Risk of bias across studies | 15 | Specify any assessment of risk of bias that may affect the cumulative evidence (e.g., publication bias, selective reporting within studies). | NA |
| Additional analyses | 16 | Describe methods of additional analyses (e.g., sensitivity or subgroup analyses, meta-regression), if done, indicating which were pre-specified. | 9 |
| **RESULTS** | | |  |
| Study selection | 17 | Give numbers of studies screened, assessed for eligibility, and included in the review, with reasons for exclusions at each stage, ideally with a flow diagram. | 9, additional file 3, figure 1 |
| Study characteristics | 18 | For each study, present characteristics for which data were extracted (e.g., study size, PICOS, follow-up period) and provide the citations. | 9-10 |
| Risk of bias within studies | 19 | Present data on risk of bias of each study and, if available, any outcome level assessment (see item 12). | 11 |
| Results of individual studies | 20 | For all outcomes considered (benefits or harms), present, for each study: (a) simple summary data for each intervention group (b) effect estimates and confidence intervals, ideally with a forest plot. | Table 2 |
| Synthesis of results | 21 | Present results of each meta-analysis done, including confidence intervals and measures of consistency. | 11-16 |
| Risk of bias across studies | 22 | Present results of any assessment of risk of bias across studies (see Item 15). | 16 |
| Additional analysis | 23 | Give results of additional analyses, if done (e.g., sensitivity or subgroup analyses, meta-regression [see Item 16]). | NA |
| **DISCUSSION** | | |  |
| Summary of evidence | 24 | Summarize the main findings including the strength of evidence for each main outcome; consider their relevance to key groups (e.g., healthcare providers, users, and policy makers). | 16 |
| Limitations | 25 | Discuss limitations at study and outcome level (e.g., risk of bias), and at review-level (e.g., incomplete retrieval of identified research, reporting bias). | 18 |
| Conclusions | 26 | Provide a general interpretation of the results in the context of other evidence, and implications for future research. | 18-20 |
| **FUNDING** | | |  |
| Funding | 27 | Describe sources of funding for the systematic review and other support (e.g., supply of data); role of funders for the systematic review. | 21 |

*From:*  Moher D, Liberati A, Tetzlaff J, Altman DG, The PRISMA Group (2009). Preferred Reporting Items for Systematic Reviews and Meta-Analyses: The PRISMA Statement. PLoS Med 6(7): e1000097. doi:10.1371/journal.pmed1000097

For more information, visit: **www.prisma-statement.org**.

Page 2 of 2

### Additional file 2 Search strategy

| **Systematic reviews** | |
| --- | --- |
| **MEDLINE**  PubMed  April 2019 | #1 “Guideline Adherence”[Mesh]  #2 adherence[ti]  #3 implement*[ti]  #4 uptake[ti]  #5 complian*[ti]  #6 #1 OR #2 OR #3 OR #4 OR #5  #7 guideline*[tiab]  #8 recommendation*[ti]  #9 #7 OR #8  #10 #6 AND #9  #11 systematic[sb]  #12 framework*[tiab]  #13 #11 OR #12  #14 #10 AND #13 |
| **EMBASE**  Ovid Embase  <1980 to 2017 Week 38>  April 2019 | #1 adherence.ti.  #2 implement*.ti.  #3 uptake.ti.  #4 complian*.ti.  #5 #1 or #2 or #3 or #4  #6 guideline*.ti,ab.  #7 recommendation*.ti.  #8 #6 or# 7  #9 #5 and #8  #10 limit #9 to "systematic review"  #11 framework*.ti,ab.  #12 #9 and #11  #13 #10 or #12 |
| **Primary studies** | |
| **MEDLINE**  PubMed  April 2019 | #1 “Guideline Adherence”[Mesh]  #2 adherence[ti]  #3 implement*[ti]  #4 uptake[ti  #5 complian*[ti]  #6 #1 OR #2 OR #3 OR #4 OR #5  #7 guideline*[tiab]  #8 recommendation*[ti]  #9 #7 OR #8  #10 #6 AND #9  #11 Neoplasms”[Majr]  #12 cancer[tiab]  #13 oncolog*[tiab]  #14 mammogram*[tiab  #15 breast[ti] AND screen*[ti]  #16 #11 OR #12 OR #13 OR #14 OR #15  #17  #10 AND #16 |
| **EMBASE**  Ovid Embase  <1980 to 2017 Week 38>  April 2019 | #1 adherence.ti.  #2 implement*.ti.  #3 uptake.ti.  #4 complian*.ti.  #5 #1 or# 2 or #3 or #4  #6 guideline*.ti,ab.  #7 recommendation*.ti.  #8  #6 or #7  #9 #5 and 8  #10 *neoplasm/  #11 cancer.ti,ab.  #12 oncolog*.ti,ab.  #13 mammogram*.ti,ab.  #14 (breast adj4 screen*).ti  #15 #10 or #11 or #12 or #13 or #14  #16 #9 and #15 |
|  |  |

### Additional file 3. Reasons for exclusion after full-text examination

| **Systematic reviews** | |
| --- | --- |
| **Study ID** | **Reason for exclusion** |
| 1. Ackermann 1994([1](#_ENREF_1)) | Not focused on the impact of guideline adherence on health outcomes |
| 1. Brouwers 2011([2](#_ENREF_2)) | Not focused on the impact of guideline adherence on health outcomes |
| 1. Eniu 2008 ([3](#_ENREF_3)) | Not focused on the impact of guideline adherence on health outcomes |
| 1. Gandhi 2015([4](#_ENREF_4)) | Not focused on the impact of guideline adherence on health outcomes |
| 1. Gluck 2010([5](#_ENREF_5)) | Not focused on the impact of guideline adherence on health outcomes |
| 1. Harford 2008([6](#_ENREF_6)) | Not focused on the impact of guideline adherence on health outcomes |
| 1. Henry 2014([7](#_ENREF_7)) | Not focused on the impact of guideline adherence on health outcomes |
| 1. Sabatino 2008([8](#_ENREF_8)) | Not focused on the impact of guideline adherence on health outcomes |
| 1. Shyyan 2008([9](#_ENREF_9)) | Not focused on the impact of guideline adherence on health outcomes |
| 1. Yip 2008([10](#_ENREF_10)) | Not focused on the impact of guideline adherence on health outcomes |
| **Individual studies** |  |
| **Study ID** | **Reason for exclusion** |
| 1. Acuna 2017 ([11](#_ENREF_11)) | Not EU country |
| 1. Beaber 2018 ([12](#_ENREF_12)) | Not EU country |
| 1. Bell 2000([13](#_ENREF_13)) | No relevant outcome examined |
| 1. Bouaud 2001([14](#_ENREF_14)) | No relevant outcome examined |
| 1. Bouaud 2002([15](#_ENREF_15)) | No relevant outcome examined |
| 1. Bouaud 2014([16](#_ENREF_16)) | No relevant outcome examined |
| 1. Bouaud 2015([17](#_ENREF_17)) | No relevant outcome examined |
| 1. Castello 2015([18](#_ENREF_18)) | Not focused on providers' adherence to CGs, but rather on patients' adherence to medical recommendations for breast cancer prevention |
| 1. Craft 2000([19](#_ENREF_19)) | Not EU country |
| 1. DeSnyder2018([20](#_ENREF_20)) | Not EU country |
| 1. Dull 2017([21](#_ENREF_21)) | Not EU country |
| 1. Eccher 2014([22](#_ENREF_22)) | No relevant outcome examined |
| 1. Gray 2018([23](#_ENREF_23)) | Not EU country |
| 1. Groot 2009([24](#_ENREF_24)) | No relevant outcome examined |
| 1. Hallowell 2018([25](#_ENREF_25)) | Not EU country |
| 1. Hamood 2019([26](#_ENREF_26)) | Not EU country |
| 1. Hofvind 2007([27](#_ENREF_27)) | Adherence of a screening program to EU guidelines, rather than healthcare providers' adherence to CGs. |
| 1. Jackisch 2008([28](#_ENREF_28)) | No relevant outcome examined |
| 1. Jensen 2005([29](#_ENREF_29)) | Not focused on CGs, but rather on quality assurance guidelines |
| 1. Katz 2018([30](#_ENREF_30)) | Not EU country |
| 1. Lane 1999([31](#_ENREF_31)) | Not EU country |
| 1. Lu 2019([32](#_ENREF_32)) | Not EU country |
| 1. Luctkar-Flude 2018 ([33](#_ENREF_33)) | Not EU country |
| 1. Palazzi 2002([34](#_ENREF_34)) | No relevant outcome examined |
| 1. Patrick 2017([35](#_ENREF_35)) | Not EU country |
| 1. Press 2018([36](#_ENREF_36)) | Not EU country |
| 1. Radhakrishnan 2018([37](#_ENREF_37)) | Not EU country |
| 1. Saldanha 2011([38](#_ENREF_38)) | Adherence to guidelines not examined |
| 1. Seroussi 2001([39](#_ENREF_39)) | No relevant outcome examined |
| 1. Seroussi 2007([40](#_ENREF_40)) | No relevant outcome examined |
| 1. Seroussi 2013([41](#_ENREF_41)) | No relevant outcome examined |
| 1. Veerbeek 2011([42](#_ENREF_42)) | No relevant outcome examined |
| 1. Young 2007([43](#_ENREF_43)) | No relevant outcome examined |
| 1. Watson 2019([44](#_ENREF_44)) | Patients adherence rather than provider adherence |

### Additional file 4 Characteristics of included studies by design

| **Study ID^1^** | **References** | **Aim** | **Country** | **Study design** | **Source of data** | **Time of data collection** | | **Guideline scope** | **Participants / characteristics** | **Outcomes** |
| --- | --- | --- | --- | --- | --- | --- | --- | --- | --- | --- |
| OncoDoc2 | ([45](#_ENREF_45)) | To propose a description of the reasons that lead clinicians not to comply with OncoDoc2 recommendations by revisiting the 4-dimension conceptual model of EBM decision of Haynes et al. | France | Case study | Medical records Oncodoc2 | Feb. 2007 – Sep. 2009 | Treatment | | 1889 medical decisions regarding the management of patients with breast cancer of MTB decisions with OncoDoc2 | Factors that impact on adherence |
|  | ([46](#_ENREF_46)) | To characterise the patients for which physicians decide not to follow CGs. |  |  |  |  |  |  |  | Factors that impact on adherence |
|  | ([47](#_ENREF_47)) | To evaluate the compliance rate of multidisciplinary tumour boards (MTB) breast cancer therapeutic decisions with CGs and to determine patient factors still associated with non-compliance. |  |  |  |  |  |  |  | Overall adherence to guidelines |
| Van Ryckeghem 2019 | ([48](#_ENREF_48)) | To describe the use of primary prophylaxis with granulocyte colony-stimulating factors in patients receiving chemotherapy for breast cancer in Belgium. | Belgium | Cross-sectional | Medical records | 2014 | Preventive therapies | | 490 breast cancer patients of any stage who were scheduled to receive myelosuppressive chemotherapy in the outpatient setting | Adherence to guidelines recommendations |
| Aristei 2008 | ([49](#_ENREF_49)) | To describe the Italian radiotherapy practice after breast-conserving surgery for early breast cancer and assess adherence to national and international guidelines. | Italy | Cross-sectional | Providers self-reported adherence | 2003-2004 | Treatment | | 66/138 (48%) Radiation oncology centres who answered a questionnaire | Adherence to guidelines recommendations |
| Barni 2011 | ([50](#_ENREF_50)) | To evaluate the compliance with guidelines in a nationwide program. | Italy | Cross-sectional | Medical records | Oct. 2005-Nov. 2006 | Diagnosis, Treatment, and follow-up | | 221 patients with clinical stage I-II invasive breast cancer age ≤70 years Sample of oncology centres | Adherence to guidelines recommendations (with strength)) |
| Natoli 2014 | ([51](#_ENREF_51)) | To investigate, through a web-based survey, surveillance methodologies selected by Italian oncologists in everyday clinical practice. | Italy | Cross-sectional | Providers self-reported adherence | Jan.-May 2013 | Follow-up | | 125/233 (53.6%) Medical Oncology Units | Adherence to guidelines recommendations |
| DURTO 2003 | ([52](#_ENREF_52)) | To investigate if antiemetic guidelines for the prevention of both acute and delayed emesis induced by highly moderately emetogenic chemotherapy were transferred in daily clinical practice in Italy 2 years after their publication. | Italy | Cross-sectional | Medical records (audit) | 16 to 23 March 2000 | Treatment prevention acute emesis during CT | | 768 consecutive breast cancer patients who received any cycle of adjuvant chemotherapy in 87 Oncological centres768 consecutive breast cancer patients who received any cycle of adjuvant chemotherapy in 87 Oncological centres | Adherence to guidelines recommendations |
|  |  |  |  |  |  |  |  |  |  |  |
| Roila 2003 | ([53](#_ENREF_53)) | To audit the prescriptions of both adjuvant chemotherapy and endocrine therapy administered to Italian breast cancer patients compared to international recommendations. | Italy | Cross-sectional | Medical records (audit) | 16 to 23 March 2000 | Treatment endocrine therapy | |  | Adherence to guidelines recommendations |
| Ray-Coquard 2012 | ([54](#_ENREF_54)) | To evaluate the adherence to the guidelines for the use of erythropoiesis-stimulating agents (ESAs) in the management of chemotherapy-induced anaemia for patients with advanced breast and lung cancers. | France | Cross-sectional | Hospital database | 2010 | Treatment (chemotherapy-induced anaemia) | | 185 breast cancer patients that had received an ESA during chemotherapy Metastatic breast cancer patients with CT | Adherence to CGs recommendations |
| Lebeau 2011 | ([55](#_ENREF_55)) | To measure compliance with CGs for non-metastatic BC care management and to identify factors associated with non-compliance at clinical and organisational levels. | France | Cross-sectional | Medical records | 2003-2004 | Treatment Diagnosis | | Nine hundred twenty-six women with invasive unilateral BC without distant metastases and at least two contacts with one of the two regional healthcare systems in the first year after diagnosis. | Overall treatment sequence adherence (clinical decisions and procedures) and by type of treatment recommendations |
| Liebrich 2011 | ([56](#_ENREF_56)) | To determine the extent to which guidelines are adopted in Germany, particularly in older patients, and to understand whether the standards required in certified breast cancer clinics are met more generally in routine clinical practice. | Germany | Cross-sectional | Online voluntary register | 2007 - 2009 | Treatment (adjuvant trastuzumab) | | 5397 patients first diagnosed with breast cancer registered cases in ONkeyLINE | Adherence to guidelines recommendations |
| de Bock 1999 | ([57](#_ENREF_57)) | To ascertain how often patients, seek help for familial breast cancer in primary care, and to identify GPs management of these patients, in order to see whether guidelines are followed. | UK | Cross-sectional | Providers self-reported adherence | 1996-1997 | Preventive Surveillance of women with breast cancer in the family | | 202 GPs in a postgraduate education programme | Adherence to CGs recommendations |
| Mylvaganam 2018 | ([58](#_ENREF_58)) | To explore the degree of uptake of best-practice CGs within units performing implant-based reconstruction as the first phase of the implant Breast Reconstruction Evaluation study | UK | Cross-sectional | Providers self-reported adherence | 2014 | Preventive therapies on surgery | | Eighty-one breast surgical and plastic units from both high and low volume centres with participating units performing a median of 35 implant-based reconstructions per year (range 0-230). | Adherence to CGs recommendations |
| Grandjean 2012 | ([59](#_ENREF_59)) | To evaluate adherence with follow-up criteria as suggested by the national guideline for breast cancer patients. | The Netherlands | Cross-sectional | Medical records of the Netherlands Cancer Registry | 2003 | Follow-up | | 196 invasive breast cancer patients and curatively treated using surgery. | Adherence to guidelines recommendations |
| Vercauteren 2010 | ([60](#_ENREF_60)) | To assess the adherence to a guideline for additional breast ultrasonography in a cross-sectional survey among hospitals in the Netherlands. | The Netherlands | Cross-sectional | Medical records | 2004 | Diagnosis | | 6457 patients referred for mammography to the radiology departments for 2 months 17 Nationwide hospitals | Adherence to CGs recommendations |
| Mille 2000 | ([61](#_ENREF_61)) | To evaluate the economic impact of changing medical practices, which are evolving toward CGs compliance, in the post-therapeutic follow-up of patients with localised breast cancer. | France | Non controlled before-after study | Medical records | 1993 and 1995 | Follow-up | | 200 patient records were chosen at random patients with localised breast cancer after the end of treatment. | Adherence to CGs recommendations |
| Ray-Coquard 1997 | ([62](#_ENREF_62)) | To assess the impact of CGs on the management of breast and colon cancer. | France | Non controlled before-after study | Medical records | 1993 and 1995 | Treatment | | 100 women with localised breast cancer randomly selected | Overall treatment sequence guideline adherence |
| Jacke 2015 | ([63](#_ENREF_63)) | To assess guideline adherence according to time intervals and its impact on survival. | Germany | Non controlled before-after study | Medical records and Hospital database | 1996-1997 and 2003-2004 | Treatment | | 877 patients with primary BC treatment were assessed 104 quality indicators overall binary adherence index in two cohort | Adherence to CGs recommendations |
| Sacerdote 2013 | ([64](#_ENREF_64)) | To evaluate the impact of regional guidelines (Piedmont guidelines) for breast cancer diagnosis and treatment on quality-of-care indicators in the North-western Italian region of Piedmont. | Italy | Non controlled before-after study | Medical records | 2002 and 2004 | Diagnosis and treatment | | Two samples of women aged 50–69 years with incident breast cancer: 600 in 2002 and 621 in 2004. | Adherence to CGs recommendations |
| Ottevanger 2004 | ([65](#_ENREF_65)) | To compare guideline adherence between the two periods, 1988–1992 and 1996–1998 for the unchanged advice to assess the effect of the audit and feedback and educational activities between these periods. | The Netherlands | Non controlled before-after study | Medical records | 1988-1992 (P1) and 1996-1998 (P2) | Treatment | | 323 premenopausal node-positive breast cancer patients in P1 and 155 patients in P2 Cancer Centre | Adherence to CGs recommendations |
| Holm-Rasmussen 2017 | ([66](#_ENREF_66)) | To investigate differences among Danish department’s adherence to the national guidelines with regards to the use of sentinel lymph node biopsy (SLNB) in DCIS patients and to identify the factors associated with the use of SLNB. | Denmark | Retrospective cohort | Registry of Danish Breast Cancer Group database | 2004-2015 | Treatment | | 2618 patients with a pre-operative diagnosis of DCIS on core needle biopsy or on excision biopsy in combination with a final postoperative diagnosis of DCIS | Adherence to guideline recommendations for surgery, overall and by subgroups of patients |
| Jensen 2018 | ([67](#_ENREF_67)) | To describe the plurality of modifications introduced over the past 10 years in the national Danish guidelines for the management of early breast cancer. | Denmark | Retrospective cohort | National database | 2007-2016 | Treatment | | 48772 patients with first invasive breast tumour registered in the clinical database of the Danish Breast Cancer Cooperative Group. | Adherence for guideline recommendations |
| Andreano 2017 | ([68](#_ENREF_68)) | To investigate the effect of adherence to CGS on long-term survival for a cohort of Italian breast cancer patients. | Italy | Retrospective cohort | Registry of Milan | 2007-2012 | Diagnosis and treatment | | 6333 incident breast cancer cases not metastatic at diagnosis receiving primary surgery | Adherence to guidelines and survival estimates. |
| Bucchi 2009 | ([69](#_ENREF_69)) | To compare screen-detected breast cancer patients with symptomatic patients for (1) the prognostic profile, (2) the total rate of adjuvant chemo/hormone therapy, and (3) the crude and adjusted likelihood of being treated according to guidelines. | Italy | Retrospective cohort | Medical records from the Romagna Cancer Registry | 1997–2001 | Treatment | | 598 (59% screen-detected) node-negative high-risk patients  430 (40% screen-detected) node-positive patients aged 50–69 years | Adherence to guideline recommendations by symptomatic or screen-detected |
| Balasubramanian 2003 | ([70](#_ENREF_70)) | To check the compliance with the guidelines eight months after the introduction of revised adjuvant treatment guidelines in breast cancer management | UK | Retrospective cohort | Medical records The North Trent Cancer Network | 2003 | Treatment | | 194 patients with invasive breast cancer who underwent definitive surgery (in 3 months) | Adherence to guideline recommendations |
| Poncet 2009 | ([71](#_ENREF_71)) | To analyse adherence to prescribing guidelines of anti-HER2 monoclonal antibody trastuzumab treatment for metastatic breast cancer. Efficacy and costs were also evaluated. | France | Retrospective cohort | Medical records Four healthcare centres | 2003-2004 | Treatment | | 131 patients with metastatic breast cancer receiving trastuzumab treatment | Adherence to guideline recommendations |
| Schrodi 2015 | ([72](#_ENREF_72)) | To describe the 12-year trend of primary surgery following the introduction of national guidelines for the management of breast cancer in Germany. | Germany | Retrospective cohort | Medical records Four different regions | 1999-2010 | Treatment Surgery | | 72742 breast cancer patients | Guideline adherence to quality indicators: Rates of BCS for pT1/2-tumours Rates of sentinel lymph node biopsy (SLNB) |
| Wimmer 2019 | ([73](#_ENREF_73)) | To assess adherence to guidelines, long-term survival, recurrence rates, and recurrence-free survival after adjuvant RT in patients with BCT in daily clinical practice. | Germany | Retrospective cohort | Population-based registry of Upper Palatinate and Lower Bavaria, Germany | 2003-2013 | Treatment. | | 6370 patients with invasive non-metastatic breast cancer of tumour stage I, II, and III and breast-conserving therapy | Adherence to guideline recommendations |
| BRENDA I | Wökel 2010(a) ([74](#_ENREF_74))(a) | To investigate the influence of the national guideline on the outcome for breast cancer patients, including data from a period of the last 13 years. | Germany | Retrospective cohort | Hospital databases | 1992–2005 | Treatment | | 2231 patients with primary breast cancer BRENDA project I | Adherence to guideline recommendations |
|  | Hancke 2010 ([75](#_ENREF_75)) | To examine the extent to which non-adherence to treatment guidelines occurs for women aged ≥70 years and changes overall survival and disease-free survival. | Germany | Retrospective cohort | Hospital databases | 1992–2005 | Treatment | | 1922 women aged ≥50 years with histologically confirmed invasive breast cancer. BRENDA project I | Adherence to guideline recommendations |
|  | Varga2010 ([76](#_ENREF_76)) | To evaluate the association between guideline-adherent vs non-adherent treatment on recurrence-free survival and overall survival in early-onset breast cancer patients. | Germany | Retrospective cohort | Hospital databases | 1992–2005 | Treatment | | 1778 patients ≤ 55y (early-onset breast cancer patients) BRENDA project I | Adherence to guideline recommendations |
|  | Wöckel 2014 ([77](#_ENREF_77)) | To answer if guideline-adherent radiotherapy improves primary breast cancer patient survival. | Germany | Retrospective cohort | Hospital databases | 1992-2008 | Treatment | | 8935 primary breast cancer patients BRENDA project I | Adherence to guideline recommendations |
|  | Van Ewijk 2015([78](#_ENREF_78)) | To examine the extent to which guideline-adherent adjuvant treatment is an equal alternative for elderly patients aged 65–80 | Germany | Retrospective cohort | Hospital databases | 1992-2008 | Treatment | | 4142 breast cancer patients (elderly patients) patients aged 65-80 participants and non-participants in RCT BRENDA project I | Adherence to guideline recommendations |
|  | Schwentner 2013([79](#_ENREF_79)) | To evaluate possible differences in survival in triple-negative breast cancer patients by age and by the extent to which evidence-based treatment guidelines have adhered. | Germany | Retrospective cohort | Hospital databases | 1992-2008 | Treatment | | 10897 in the database9156 primary breast cancer patients with TNBC Her2 status available BRENDA project I | Adherence to guidelines recommendations by tumour characteristics |
|  | Ebner 2015 ([80](#_ENREF_80)) | To investigate the associations among tumour characteristics, guideline adherence, and outcomes; and to compare these associations between younger and older breast cancer patients. | Germany | Retrospective cohort | Hospital databases | 1992-2008 | Treatment | | 10897 in the database7732 women aged ≥50 years with histologically confirmed invasive breast cancer BRENDA project I. | Adherence to guidelines recommendations |
|  | Ebner 2015(a)([81](#_ENREF_81))(a) | To investigate correlations between the biological subgroups. | Germany | Retrospective cohort | Hospital databases | 1992-2008 | Treatment | | 10897 in the database5632 primary breast cancer patients with complete tumour information BRENDA project I | Adherence to guidelines recommendations |
|  | Wollschlager 2017 ([82](#_ENREF_82)) | To investigate the association between baseline comorbidity and age with the administration of surgery, chemotherapy, radiotherapy, and endocrine therapy, to investigate the impact of guideline adherent adjuvant treatment on survival in comorbid patients. | Germany | Retrospective cohort | Hospital databases | 1992-2008 | Treatment | | 10897 in the database from one hospital collected the Charlson Comorbidity index 2137 primary breast cancer patients BRENDA project I | Guideline adherence to adjuvant treatment recommendations |
|  | Wolters 2015 ([83](#_ENREF_83)) | To resolve the pseudo-paradox that the clinical outcome of women affected by breast cancer has improved during the last 20 years, irrespective of whether they were treated following CGs or not. | Germany | Retrospective cohort | Hospital databases | 1991 -2009 | Treatment | | 9061 primary breast cancer patients, comparing 1991-200 and 2001-2009 survival rates BRENDA project I | Overall guideline adherence (surgery, RT, ET, CT) |
|  | Wöckel 2010 ([84](#_ENREF_84)) | To analyse the impact of German-S3-breast cancer guideline adherence on clinical outcomes and certified centres. | Germany | Retrospective cohort | Hospital databases | 2001-2005 | Treatment | | 3976 patients with primary breast cancer BRENDA project I | Adherence to guidelines recommendations |
|  | Schwentner 2012(a) ([85](#_ENREF_85)) | To investigate: (1) Is there an impact of guideline-adherent treatment on RFS and OAS in TNBC? (2) Which adjuvant treatment has the most critical impact on RFS and OAS in TNBC? | Germany | Retrospective cohort | Hospital databases | 2000-2005 | Treatment | | 3658 primary breast cancer patients BRENDA project I | Adherence to guidelines recommendations by specific subgroups |
|  | Schwentner 2012(b) ([86](#_ENREF_86))(b) | To examine survival parameters in patients with bilateral vs unilateral unifocal breast cancer, and to examine treatment patterns and the influence of guideline adherence on survival. | Germany | Retrospective cohort | Hospital databases | 2000-2005 | Treatment | | 5292 primary breast cancer patients- with 229 (4.3%) patients with bilateral breast cancer BRENDA project I | Adherence to guidelines recommendations |
| Schreuder 2017 | ([87](#_ENREF_87)) | to evaluate the clinical implications (CT administration) of gene expression profile (GEP) use (MammaPrint 70-gene signature) and GEP test results when used outside the guideline intended GEP indication area. | The Netherlands. | Retrospective cohort | Medical records from the Netherlands Cancer Registry | 2011-2014 | Treatment | | 26425 patients for whom the current Dutch treatment guidelines state a clear advice to administer or withhold CT, so without an indication to perform a GEP | The factor that impacts on adherence |
| Schaapveld 2004 | ([88](#_ENREF_88)) | To review the diagnostic procedures and cytotoxic treatment of node-positive breast cancer patients diagnosed from January 1993 to January 1996 to evaluate the implementation of and compliance with the regional treatment guidelines. | The Netherlands | Retrospective cohort | Cancer registry | 1993-1996 | Treatment | | 251 consecutive axillary lymph node-positive breast cancer patients <50 years old from the North area | Adherence to guidelines recommendations |
| Schaapveld 2005 | ([89](#_ENREF_89)) | To analyse variations in surgical treatment and guideline compliance concerning the application of radiotherapy and axillary lymph node dissection (ALND), for early breast cancer, before and after the sentinel node biopsy (SLNB) introduction. | The Netherlands | Retrospective cohort | Cancer registry | 1989-2002 | Treatment | | 13532 consecutive surgically treated stage I–IIIA breast cancer patients North | Adherence to guidelines recommendations |
| de Roos 2005 | ([90](#_ENREF_90)) | To study the effect of compliance with guidelines on local recurrence (LR)-free survival in patients treated for ductal carcinoma in situ (DCIS). | The Netherlands | Retrospective cohort | Cancer registry | 1992 2003 | Treatment | | Two hundred fifty-one consecutive patients treated for ductal carcinoma in situ (DCIS) in the period. Two hospitals North | Adherence to guidelines recommendations |
| Weggelaar 2011 | ([91](#_ENREF_91)) | To evaluate the adherence to a consensus protocol in unselected women aged 80 years and older in the Netherlands. | The Netherlands | Retrospective cohort | Cancer registry | 2001-2006 | Treatment Surgical | | 2336 female patients, 60 years and older, diagnosed with breast cancer TNM stage I–IIIa from the East regions | Adherence to guidelines recommendations |
| Visser 2016 | ([92](#_ENREF_92)) | To gain insight into left ventricular ejection fraction (LVEF) monitoring during adjuvant trastuzumab treatment in clinical practice. | The Netherlands | Retrospective cohort | Cancer registry | 2006-2011 | Preventive monitoring trastuzumab | | 328 female patients diagnosed with a HER2 positive breast from 3 hospitals | Adherence to guidelines recommendations |
| de Munck 2011 | ([93](#_ENREF_93)) | To identify the number of women with HER2-positive breast cancer and to evaluate the level of implementation of adjuvant trastuzumab in clinical practice. | The Netherlands | Retrospective cohort | National Cancer registry | 2005-. 2007 | Treatment trastuzumab treatment | | 14934 women diagnosed with primary breast cancer. | Adherence to guidelines recommendations |
| van de Water 2012 | ([94](#_ENREF_94)) | To assess adherence to treatment guidelines by age at diagnosis, and to examine age-specific survival about adherence to guidelines. | The Netherlands | Retrospective cohort | National Cancer registry | 2005 - 2008 | Treatment | | 31520 early-stage breast cancer women | Adherence to guidelines recommendations and by age subgroups (≥75 y and <65 y) |
| Kuijer 2017 | ([95](#_ENREF_95)) | To assess whether socioeconomic status and ethnicity affect adjuvant systemic therapy guideline adherence in early breast cancer patients in a health care setting with assumed equal access to care. | The Netherlands | Retrospective cohort | National Cancer registry | 2005 - 2014 | Treatment | | 104201 women surgically treated for primary, unilateral, invasive breast cancer | Adherence to guidelines recommendations |
| Heins 2017 | ([96](#_ENREF_96)) | To study the level of adherence to treatment recommendations made in Dutch national cancer treatment guidelines, and factors affecting this adherence. | The Netherlands | Retrospective cohort | Cancer Registry | 2007-2012 | Treatment | | Non-reported. Hormone therapy was measured in women with Stage II/III, age>70, ER/PR+ and surgery | Adherence to guideline recommendations for Endocrine therapy and Chemotherapy |
| BRENDA II | Schwentner 2016 ([97](#_ENREF_97)) | To examine patient- and physician-related factors that influence therapy decisions that prevent patients from undergoing guideline-adherent adjuvant treatment in primary breast cancer care. | Germany | Prospective cohort | Medical records | 2009-2012 | Treatment | | 857, diagnosed with primary, histologically confirmed breast cancer within the BRENDA II project | Adherence to guidelines recommendations |
|  | Stuber2017([98](#_ENREF_98)) | To examine factors that might influence patients’ and physicians’ decisions against the initiation of guideline adherent adjuvant endocrine therapy. | Germany | Prospective cohort | Medical records | 2009-2012 | Treatment | | 857, diagnosed with primary, histologically confirmed breast cancer within the BRENDA II project | Adherence to guidelines recommendations |
|  | Leinert 2019 ([99](#_ENREF_99)) | To examine the association between cognitive impairment and guideline adherence for application of chemotherapy in older patients with breast cancer. | Germany | Prospective cohort | Medical records | 2009-2012 | Treatment | | 263, patients aged ≥65 years with primary breast cancer non-metastatic or recurrent disease at baseline within the BRENDA II project | Adherence to guidelines recommendations |
| Boskovic 2017 | ([100](#_ENREF_100)) | To investigate adherence to vitamin D and calcium in postmenopausal breast cancer patients receiving adjuvant non-steroidal aromatase inhibitors, and oncologists' adherence to the bone health guidelines. | Croatia | Prospective cohort | Medical records | 2017 | Preventive therapy | | 438 newly diagnosed patients and those who have already been receiving non-steroidal aromatase inhibitors for up to 3.5 years | Adherence to guidelines recommendations |
| Smith 2016 | ([101](#_ENREF_101)) | To investigate the factors affecting the implementation of preventive therapy within the UK. | United Kingdom | Qualitative study | Interviews Providers perceptions | 2013 | Preventive therapy | | Ten general practitioners and 15 clinicians are working in family history or clinical genetics settings. | Clinicians’ perceptions of factors that impact on adherence |

^1^ References are sorted by type study design, and within each one by country and time of data collection. ALND, axillary lymph node dissection; CGs, clinical guidelines; CT, chemotherapy; DCIS, ductal carcinoma in situ; ESAs, erythropoiesis-stimulating agents; GEP, gene expression profile; GPs, general practitioners; LVEF, left ventricular ejection fraction; LR, local recurrence; MTB, multidisciplinary tumour board; OS, overall survival; RT, radiotherapy; RFS, recurrence-free survival; SLNB, sentinel lymph node biopsy; TNBC, triple-negative breast cancer.

**Additional file 5. Reporting of methods to measure adherence**

| **Reference** | **Adherence Measurement** |
| --- | --- |
| Andreano 2017([102](#_ENREF_102)) | We defined as adherent the care pathway of patients with a proportion of met indicators equal or greater than 80%” “Sensitivity analyses were performed considering different cut-offs of adherence (from 60 to 90% by 10%)” |
| Balasubramanian 2003([70](#_ENREF_70)) | Compliance was defined as the ratio of the number of decisions that agreed with the guidelines to the total number of decisions where eligibility for adjuvant treatment could be ascertained from the records; this was expressed as a percentage |
| Barni 2011 ([50](#_ENREF_50)) | A summary indicator, representing the overall proportion of cases in which guidelines were correctly employed, was also calculated as the ratio between the number of patients who had been treated following guidelines and the total number of eligible patients for each indicator. Nine indicators were used to verify an agreement between guidelines and practice in clinical stage I and II invasive breast cancer. |
| Boskovic 2017([100](#_ENREF_100)) | Adherence to specific guidelines recommendations (rather than overall adherence) was calculated: Percentage of patients receiving densitometry before initiation of AIs therapy. Percentage of patients receiving vitamin D and calcium, and bisphosphonate therapy where indicated. The Patients who took 80% of the prescribed dose of the vitamin D and calcium were considered adherent |
| BRENDA I  Ebner, Hancke et al. 2015 ([80](#_ENREF_80))  Wockel 2010 ([84](#_ENREF_84)) | The definition of evidence-based guideline-adherent adjuvant treatment was based on internationally validated guidelines. We decided to base the definition of guideline-adherent adjuvant treatment on the German national consensus guideline (S3-guideline) for the decision of loco-regional treatment (surgery, radiotherapy), for chemotherapy and endocrine therapy. All applied therapy regimens were retrospectively evaluated concerning their adherence to the S3-guideline (103). The omission of any suggested adjuvant treatment or abandon of any adjuvant treatment was classified as non-attending the suggested adjuvant therapy. Therefore, the adjuvant therapy is divided into subgroups (operation of the breast, axillary lymph node dissection, chemotherapy and radiotherapy). Ebner, Hancke et al. 2015 (80) Adherence to the German national consensus guideline was defined as GL+. According to Wolters et al. (103), international treatment recommendations (valid to 2011) are identical and differ only marginally in adjuvant endocrine therapy. Treatment forms were classified as GL- if GL+ was not undertaken. Likewise, the treatment subgroups (operation on the breast, axillary lymph node dissection, chemotherapy, endocrine therapy, and radiotherapy) were defined as GL+. |
| BRENDA II Schwentner 2016 ([97](#_ENREF_97)) | Adherence to the initial treatment decision was established by comparing the treatment decision, taken by the tumour board (TB) and documented by physicians, with the subsequently received CT |
| Bucchi 2009([69](#_ENREF_69)) | Compared the observed treatment with the applicable guidelines, assuming that all patients were menopausal, hormone therapy was the standard treatment for node-negative high-risk patients and node-positive patients with ER/ PgR status unknown, hormone therapy plus CT but no ET alone was the standard treatment for node-negative ER/PgR positive patients in 1998 and 2001 guidelines, and any pattern of treatment was adequate for patients enrolled in trials of adjuvant chemo/hormone therapy. |
| de Munck 2011 ([93](#_ENREF_93)) | Percentage of women with HER2-positive breast cancer receiving trastuzumab in conjunction with adjuvant chemotherapy. |
| de Roos. 2005 ([90](#_ENREF_90)) | Compliance with guidelines was stated as follows: treatment was classified as appropriate (guidelines +) if the interventions are undertaken were in agreement with the guidelines in operation at the time of treatment, whereas deviations from the guidelines were classified as inappropriate (guidelines -). Owing to changes in the guidelines, the total study period is divided into three separate periods (1992–1995, 1996–1999 and 2000–2003). |
| Grandjean 2012 ([59](#_ENREF_59)) | Guideline adherence definition: Comparison of the actual performance of follow-up care with the recommendations in the national guideline. Three categories for the number of consultations and mammograms reconstructions: less than recommended; as recommended; more than recommended. Adherence rate was defined as the percentage of women receiving the specific service (consultations/mammogram) as recommended |
| Heins 2017 ([96](#_ENREF_96)) | Overall adherence to the guidelines was calculated with 15 dummy (0/1) variables for each recommendation, both at the patient and the hospital level. |
| Holm-Rasmussen 2017 ([66](#_ENREF_66)) | Sentinel lymph node biopsy (SLNB) used only where indicated. SLNB is recommended in DCIS patients undergoing mastectomy or in DCIS patients undergoing Breast Conservative Surgery when their lesion is larger than 50 mm (ultrasound or mammographic), has Van Nuys classification group III, is palpable, as well as when the DCIS lesion is located in the upper lateral quadrant of the breast |
| Jacke. 2015 ([63](#_ENREF_63)) | Cohort 1996–97 was exposed to the “free-interplay” of institutions. Primary BC treatment followed the S1-guidelines [6–9]. Cohort 2003–04 was exposed to an “integrated care” model defined by a certified BC centre. Primary BC treatment followed recommendations of the national S3-guidelines. Quality indicators (QI) operationalized guideline recommendations in two categories. First, recommendations that should be respected by physicians if all other ancillary conditions are fulfilled were one category. This QI category translated to Guideline Adherent Decisions (GAD). Second, medical decisions against recommendations of the guidelines were defined by Guideline Divergent Decisions (GDD). It is important to note that GADs and GDDs are not always the opposite of each other Developed QIs were aggregated into four indices concerning the adherence status of every therapy sequence. However, all QIs contributed to one overall binary adherence index. The aggregation of QIs was performed by the following methodology. First, each QI was assessed according to its category (GAD, GDD). Second, if all GADs were assessed as positive (e.g., adherent), BC treatment of one patient was preliminarily considered to be guideline adherent by the summarizing overall adherence index. But, if even one GAD did not catch up with guideline recommendations, the adherence index was devalued and considered to be guideline-divergent. Third, even when one GDD was administered as positive (e.g., divergent), inpatient primary BC therapy was classified as guideline-divergent by the overall adherence index. In this sense, only one disrespected quality indicator devalued all possible guideline-adherent indicators beforehand |
| Jensen 2018 ([67](#_ENREF_67)) | The omission of axillary lymph node dissection (ALND) in 2013-2016, after the implantation of the 2011 guidelines. ALND is limited to node positive cases and cases not eligible for the sentinel node technique. HER2 assessment for all breast cancer patients in 2016, after the implantation of the 2010 guidelines. Radiotherapy (RT) fraction schedule of 50 Gy/25 Fr for patients with invasive breast cancer, who had breast only RT and not included in a randomised trial. RT registered in 2009, after the implantation of the January 2009 guidelines. Hypofractionation based on 40 Gy/15 Fr for selected patients treated with breast only RT in 2016, after the implantation of the 2010 guidelines. All patients, irrespective of laterality, receiving loco-regional Radiotherapy had the internal mammary nodes included in 2016, after the implantation of the 2014 guidelines.  Up-front letrozole to postmenopausal patients not included in a randomised trial in 2016, after the implantation of the 2009 guidelines. The recommended adjuvant chemotherapy is taxane-based (three-weekly cycles of EC (600, 90 mg/m2) followed by either three-weekly cycles of docetaxel (100 mg/m2) or nine weekly cycles of paclitaxel (80 mg/m2)) in 2016, after the implantation of the 2007 guidelines. |
| Kuijer 2017 ([95](#_ENREF_95)) | Guideline non-adherence was defined as follows: chemotherapy/endocrine therapy administration without guideline indication (defined as ‘overtreatment’) or refrain chemotherapy/endocrine therapy despite a guideline indication to administer CT/ET (‘undertreatment’). |
| Lebeau 2011 ([55](#_ENREF_55)) | Guideline adherence definition: 20 criteria were used to assess compliance with the care process for each patient. Each criterion was classified into three levels of compliance: (C) compliance with CPGs; (J) justifiable non-compliance (i.e., not strictly compliant but documented justification; (NC) non-compliance with CPGs and no justification available in the patient’s medical record. Overall treatment compliance with CPGs for each patient was defined as follows:  •Compliant treatment: if all therapeutic clinical decisions for treatment and therapeutic procedures were compliant (C) or justifiable (J);  •Non-compliant treatment if at least one of these was not compliant with standards (NC). |
| Liebrich 2011 ([56](#_ENREF_56)) | Guideline adherence definition: Percentage of patients receiving treatment according to the new guideline |
| Mille 2000 ([61](#_ENREF_61)) | Follow-up was determined to be CPG-compliant or CPG non-compliant by analysing the clinical relevance of each investigation performed. This enables to differentiate investigations that were prescribed systematically from those that were justified by the presence of warning signs. |
| Mylvaganam 2018 ([58](#_ENREF_58)) | - Compliance was defined as the approval from a ‘New Techniques and Devices’ or another appropriate clinical governance committee prior to introducing the technique for patients undergoing biological mesh-assisted reconstruction. - Compliance with the audit of the short and long term clinical, cosmetic and patient-reported outcomes of reconstructive surgery for patients undergoing biological mesh-assisted reconstruction |
| OncoDoc2 Bouaud 2011([45](#_ENREF_45)) | Adherence defined, as the proportion of medical decisions taken by multidisciplinary staff meetings is concordant with recommended care according to guidelines Intervention: OncoDoc2 is a guideline-based clinical decision support system (CDSS) providing patient-specific recommendations in the management of breast cancer. |
| Ottevanger 2004([65](#_ENREF_65)) | Overall guideline adherence not examined. Instead, adherence was measured based on individual indicators:  • percentage of patients with breast-conserving surgery and secondary surgery  •percentage of patients with more than 10 reported resected lymph nodes,  •with pathology reports reporting differentiation grade,  •with a known hormonal receptor status receiving chemotherapy, as advised in the guideline,  •who started chemotherapy within the advised 28 days after completion of the surgery,  • who completed chemotherapy within 1 week after the ideal time of completion, and  • with a dose intensity (DI) of chemotherapy of ≥85% |
| Poncet, Colin et al 2009 ([71](#_ENREF_71)) | The adherence to the trastuzumab treatment plan was analysed according to both the French post-licensing guidelines published in 2001 and the regional clinical guidelines published by the regional oncology care network called “Convergence” in the French Rhone-Alpes area |
| Ray Coquard 1997 ([62](#_ENREF_62)) | Guideline adherence definition: The main outcome was the number of medical decisions (overall treatment sequence) judged to conform to the CPGs. The decisions for each type of procedure individually (surgery, radiotherapy, chemotherapy, hormonal therapy, initial examination, follow-up) were assessed for conformity with the recommendations in the CPGs. The overall treatment sequence was judged to be compliant if all the component procedures were compliant. |
| Sacerdote 2013 ([64](#_ENREF_64)) | Fourteen quality of care indicators based on Clinical guidelines indicators chose to evaluate the impact of a clinical practice guideline on breast cancer treatment in Piedmont (data collected from medical charts). |
| Schaapveld 2005 ([89](#_ENREF_89)) | For the evaluation of guideline compliance, breast-conserving therapy was scored as ‘appropriate’ when it included an ALND and was complemented with radiotherapy; an MRM was considered in accordance with the guideline if complemented by an ALND. The omission of ALND was allowed after a negative SNB. An ALND with 43 positive nodes was considered an indication for regional radiotherapy; omission of radiotherapy was scored as ‘inappropriate’. The omission of radiotherapy for node-positive medially located tumours was also scored as ‘inappropriate’ in the evaluation of guideline adherence |
| Schrodi 2015 ([72](#_ENREF_72)) | Guideline adherence was analysed according to the process quality indicators, which are declared in the guidelines. Two quality indicators were analysed. First, according to quality indicator 12 of the 2004 guideline, more than 60% of all patients with pT1 or pT2 tumours should undergo BCS. Second, according to quality indicator 16a of the 2008 guideline, the axillary staging of patients with invasive breast cancer should be conducted with SLNB in at least 60% of all cases |
| Van Ryckeghem 2019 ([48](#_ENREF_48)) | The use of primary prophylaxis with granulocyte colony-stimulating factors (PPG) adherent to EORTC guidelines. The proportion of patients receiving PPG for chemotherapy regimens associated with a low, intermediate and high risk of FN. The EORTC definition of FN was applied: an ANC < 0.5 × 109/L, or an ANC < 1.0 × 109/L that is predicted to fall to < 0.5 × 109/L within 48 h, with fever (a temperature of 38.3 °C once or of 38.0 °C for more than 1 h) or clinical signs of sepsis or fever; the ANC should be measured on the same day as these clinical signs or feverish temperature or within 1 day of them. |
| Vercauturen 2010 ([60](#_ENREF_60)) | The adherence rates are reflecting the percentage of cases for which the radiologists are complying with the proposed recommendations. The adherence rate was calculated by dividing the number of patients referred for breast imaging who were treated by the radiologist according to the recommendations by the total number of patients referred for breast imaging |
| Visser 2016 ([92](#_ENREF_92)) | Percentage of patients how received guideline concordant monitoring (number of patients monitored/total number of patients eligible for monitoring according to guidelines) |
| Weggelaar 2011 ([91](#_ENREF_91)) | Number of patients receiving guideline-concordant treatment out of the total number of patients that would be eligible for that treatment according to guidelines |
| Wimmer 2019 ([73](#_ENREF_73)) | Use of radiotherapy after breast-conserving therapy in invasive breast cancer. |

**Additional file 6. Summary of quality assessment results of included studies**

| **Cross-sectional studies (n= 13)** | **(1)** | **Non controlled Before-After studies (n=5)** | **(2)** | **Retrospective cohort (n=19)** | **(3)** | **Prospective cohort (n=2)** | **(3)** | **Qualitative studies (n=1)** | **(4)** |
| --- | --- | --- | --- | --- | --- | --- | --- | --- | --- |
| Aristei 2008 | 7/10 | Jacke 2015 | Fair | Andreano 2017 | 9/9 | Boskovic 2017 | 9/9 | Smith 2016 | VI and VII not met |
| Barni 2011 | 8/10 | Mille 2000 | Fair | Balasubramanian 2003 | 8/9 | BRENDA II (Schwentner 2016) | 8/9 |  |  |
| de Bock 1999 | 7/10 | Ottevanger 2004 | Fair | Bucchi 2009 | 8/9 |  |  |  |  |
| DURTO 2003 | 7/10 | Ray-Coquard 1997 | Good | de Munck 2011 | 8/9 |  |  |  |  |
| Grandjean 2012 | 8/10 | Sacerdote 2013 | Good | de Roos 2005 | 8/9 |  |  |  |  |
| Lebeau 2011 | 7/10 |  |  | BRENDA I (Wökel 2010(a)) | 8/9 |  |  |  |  |
| Liebrich 2011 | 3/10 |  |  | Heins 2017 | 9/9 |  |  |  |  |
| Mylvaganam 2018 | 6/10 |  |  | Holm-Rasmussen 2017 | 8/9 |  |  |  |  |
| Natoli 2014 | 10/10 |  |  | Jensen 2018 | 9/9 |  |  |  |  |
| Ray-Coquard 2012 | 8/10 |  |  | Kuijer 2017 | 8/9 |  |  |  |  |
| Roila 2003 | 7/10 |  |  | Poncet 2009 | 5/9 |  |  |  |  |
| Van Ryckeghem 2019 | 7/10 |  |  | Schaapveld 2004 | 8/9 |  |  |  |  |
| Vercauteren 2010 | 9/10 |  |  | Schaapveld 2005 | 7/9 |  |  |  |  |
|  |  |  |  | Schreuder 2017 | 7/9 |  |  |  |  |
|  |  |  |  | Schrodi 2015 | 9/9 |  |  |  |  |
|  |  |  |  | van de Water 2012 | 8/9 |  |  |  |  |
|  |  |  |  | Visser 2016 | 8/9 |  |  |  |  |
|  |  |  |  | Weggelaar 2011 | 6/9 |  |  |  |  |
|  |  |  |  | Wimmer 2019 | 9/9 |  |  |  |  |
|  |  |  |  |  |  |  |  |  |  |
|  |  |  |  |  |  |  |  |  |  |

1. AXIS-Appraisal tool for Cross-Sectional Studies: The total quality score varied between 0 and 10 where 1-4 = (Low), 5-7 = (Moderate) and 8-10= (High);
2. Quality Assessment Tool for Before-After (Pre-Post) Studies with No Control Group: Quality Rating (Good, Fair, or Poor);
3. The Newcastle -Ottawa Quality Assessment Scale: The total score was from 0 (highest risk of bias) to 9 (lowest risk of bias).
4. CASP Critical Appraisal Skills Program Tool: I. Was there a clear statement of the aims of the research? II. Is a qualitative methodology appropriate? III. Was the research design appropriate to address the aims of the research? IV. Was the recruitment strategy appropriate to the aims of the research? V. Were the data collected in a way that addressed the research issue? VI. Has the relationship between the researcher and participants been adequately considered? VII. Have ethical issues been taken into consideration? VIII. Was the data analysis sufficiently rigorous? IX. Is there a clear statement of findings? X. How valuable was the research?

**References**

1. Ackermann SP, Cheal N. Factors affecting physician adherence to breast cancer screening guidelines. J Cancer Educ. 1994;9(2):96-100.

2. Brouwers MC, De Vito C, Bahirathan L, Carol A, Carroll JC, Cotterchio M, et al. What implementation interventions increase cancer screening rates? a systematic review. Implement Sci. 2011;6:111.

3. Eniu A, Carlson RW, El Saghir NS, Bines J, Bese NS, Vorobiof D, et al. Guideline implementation for breast healthcare in low- and middle-income countries: treatment resource allocation. Cancer. 2008;113(8 Suppl):2269-81.

4. Gandhi S, Verma S, Ethier JL, Simmons C, Burnett H, Alibhai SM. A systematic review and quality appraisal of international guidelines for early breast cancer systemic therapy: Are recommendations sensitive to different global resources? Breast. 2015;24(4):309-17.

5. Gluck S, Mamounas T. Improving outcomes in early-stage breast cancer. Oncology (Williston Park). 2010;24(11 Suppl 4):1-15.

6. Harford J, Azavedo E, Fischietto M. Guideline implementation for breast healthcare in low- and middle-income countries: breast healthcare program resource allocation. Cancer. 2008;113(8 Suppl):2282-96.

7. Henry NL, Hayes DF, Ramsey SD, Hortobagyi GN, Barlow WE, Gralow JR. Promoting quality and evidence-based care in early-stage breast cancer follow-up. J Natl Cancer Inst. 2014;106(4):dju034.

8. Sabatino SA, Habarta N, Baron RC, Coates RJ, Rimer BK, Kerner J, et al. Interventions to increase recommendation and delivery of screening for breast, cervical, and colorectal cancers by healthcare providers systematic reviews of provider assessment and feedback and provider incentives. Am J Prev Med. 2008;35(1 Suppl):S67-74.

9. Shyyan R, Sener SF, Anderson BO, Garrote LM, Hortobagyi GN, Ibarra JA, Jr., et al. Guideline implementation for breast healthcare in low- and middle-income countries: diagnosis resource allocation. Cancer. 2008;113(8 Suppl):2257-68.

10. Yip CH, Smith RA, Anderson BO, Miller AB, Thomas DB, Ang ES, et al. Guideline implementation for breast healthcare in low- and middle-income countries: early detection resource allocation. Cancer. 2008;113(8 Suppl):2244-56.

11. Acuna SA, Sutradhar R, Camacho X, Daly C, Del Giudice ME, Kim SJ, et al. Uptake of Cancer Screening Tests Among Recipients of Solid Organ Transplantation. Am J Transplant. 2017;17(9):2434-43.

12. Beaber EF, Sprague BL, Tosteson ANA, Haas JS, Onega T, Schapira MM, et al. Multilevel Predictors of Continued Adherence to Breast Cancer Screening Among Women Ages 50-74 Years in a Screening Population. J Womens Health (Larchmt). 2018.

13. Bell CM, Ma M, Campbell S, Basnett I, Pollock A, Taylor I. Methodological issues in the use of guidelines and audit to improve clinical effectiveness in breast cancer in one United Kingdom health region. Eur J Surg Oncol. 2000;26(2):130-6.

14. Bouaud J, Seroussi B, Antoine EC, Zelek L, Spielmann M. A before-after study using OncoDoc, a guideline-based decision support-system on breast cancer management: impact upon physician prescribing behaviour. Stud Health Technol Inform. 2001;84(Pt 1):420-4.

15. Bouaud J, Seroussi B. Impact of site-specific customizations on physician compliance with guidelines. Stud Health Technol Inform. 2002;90:543-7.

16. Bouaud J, Blaszka-Jaulerry B, Zelek L, Spano JP, Lefranc JP, Cojean-Zelek I, et al. Health information technology: use it well, or don't! Findings from the use of a decision support system for breast cancer management. AMIA Annu Symp Proc. 2014;2014:315-24.

17. Bouaud J, Spano JP, Lefranc JP, Cojean-Zelek I, Blaszka-Jaulerry B, Zelek L, et al. Physicians' Attitudes Towards the Advice of a Guideline-Based Decision Support System: A Case Study With OncoDoc2 in the Management of Breast Cancer Patients. Stud Health Technol Inform. 2015;216:264-9.

18. Castello A, Prieto L, Ederra M, Salas-Trejo D, Vidal C, Sanchez-Contador C, et al. Association between the Adherence to the International Guidelines for Cancer Prevention and Mammographic Density. PLoS One. 2015;10(7):e0132684.

19. Craft PS, Zhang Y, Brogan J, Tait N, Buckingham JM. Implementing clinical practice guidelines: A community-based audit of breast cancer treatment. Medical Journal of Australia. 2000;172(5):213-6.

20. DeSnyder SM, Hunt KK, Dong W, Smith BD, Moran MS, Chavez-MacGregor M, et al. American Society of Breast Surgeons' Practice Patterns After Publication of the SSO-ASTRO-ASCO DCIS Consensus Guideline on Margins for Breast-Conserving Surgery With Whole-Breast Irradiation. Ann Surg Oncol. 2018;25(10):2965-74.

21. Dull B, Linkugel A, Margenthaler JA, Cyr AE. Overuse of chest CT in patients with stage I & II breast cancer: An opportunity to increase guidelines compliance at an NCCN member institution. JNCCN Journal of the National Comprehensive Cancer Network. 2017;15(6):783-9.

22. Eccher C, Seyfang A, Ferro A. Implementation and evaluation of an Asbru-based decision support system for adjuvant treatment in breast cancer. Comput Methods Programs Biomed. 2014;117(2):308-21.

23. Gray BM, Vandergrift JL, Lipner RS. Association between the American Board of Internal Medicine's General Internist's Maintenance of Certification Requirement and Mammography Screening for Medicare Beneficiaries. Womens Health Issues. 2018;28(1):35-41.

24. Groot P, Hommersom A, Lucas PJ, Merk RJ, ten Teije A, van Harmelen F, et al. Using model checking for critiquing based on clinical guidelines. Artif Intell Med. 2009;46(1):19-36.

25. Hallowell BD, Puricelli Perin DM, Simoes EJ, Paez DC, Parra DC, Brownson RC, et al. Breast cancer related perceptions and practices of health professionals working in Brazil's network of primary care units. Prev Med. 2018;106:216-23.

26. Hamood R, Hamood H, Merhasin I, Keinan-Boker L. Hormone therapy and osteoporosis in breast cancer survivors: assessment of risk and adherence to screening recommendations. Osteoporosis International. 2019;30(1):187-200.

27. Hofvind S, Geller B, Vacek PM, Thoresen S, Skaane P. Using the European guidelines to evaluate the Norwegian Breast Cancer Screening Program. Eur J Epidemiol. 2007;22(7):447-55.

28. Jackisch C, Untch M, Chatsiproios D, Lamparter C, Overkamp F, Lichtenegger W, et al. Adherence to Treatment Guidelines in Breast Cancer Care - a Retrospective Analysis of the 'Organgruppe Mamma der Arbeitsgemeinschaft Gynaekologische Onkologie'. Breast Care (Basel). 2008;3(2):87-92.

29. Jensen A, Mikkelsen GJ, Vestergaard M, Lynge E, Vejborg I. Compliance with European guidelines for diagnostic mammography in a decentralized health-care setting. Acta Radiol. 2005;46(2):140-7.

30. Katz D, Tengekyon AJ, Kahan NR, Calderon-Margalit R. Patient and physician characteristics affect adherence to screening mammography: A population-based cohort study. PLoS ONE. 2018;13(3):e0194409.

31. Lane DS, Messina CR. Methodology for targeting physicians for interventions to improve breast cancer screening. Am J Prev Med. 1999;16(4):289-97.

32. Lu M, Spigelman AD. Adherence to referral guidelines: Genetic testing in an Australian triple negative breast cancer (TNBC) cohort. International Journal of Health Governance. 2019;24(1):6-18.

33. Luctkar-Flude M, Aiken A, McColl MA, Tranmer J. What do primary care providers think about implementing breast cancer survivorship care? Current Oncology. 2018;25(3):196-205.

34. Palazzi M, De Tomasi D, D'Affronto C, Richetti A, Valli MC, Meregalli S, et al. Are international guidelines for the prescription of adjuvant treatment for early breast cancer followed in clinical practice? Results of a population-based study on 1547 patients. Tumori. 2002;88(6):503-6.

35. Patrick JL, Hasse ME, Feinglass J, Khan SA. Trends in adherence to NCCN guidelines for breast conserving therapy in women with Stage I and II breast cancer: Analysis of the 1998-2008 National Cancer Data Base. Surgical Oncology. 2017;26(4):359-67.

36. Press DJ, Ibraheem A, Dolan ME, Goss KH, Conzen S, Huo D. Racial disparities in omission of oncotype DX but no racial disparities in chemotherapy receipt following completed oncotype DX test results. Breast Cancer Res Treat. 2018;168(1):207-20.

37. Radhakrishnan A, Nowak SA, Parker AM, Visvanathan K, Pollack CE. Linking physician attitudes to their breast cancer screening practices: A survey of US primary care providers and gynecologists. Prev Med. 2018;107:90-102.

38. Saldanha JD, Garrett RM, Snaddon L, Longmuir M, Bradshaw N, Watt C, et al. Impact of national guidelines on family history breast cancer surveillance. Scott Med J. 2011;56(4):203-5.

39. Seroussi B, Bouaud J, Antoine EC. ONCODOC: a successful experiment of computer-supported guideline development and implementation in the treatment of breast cancer. Artif Intell Med. 2001;22(1):43-64.

40. Seroussi B, Bouaud J, Gligorov J, Uzan S. Supporting multidisciplinary staff meetings for guideline-based breast cancer management: a study with OncoDoc2. AMIA Annu Symp Proc. 2007:656-60.

41. Seroussi B, Soulet A, Spano JP, Lefranc JP, Cojean-Zelek I, Blaszka-Jaulerry B, et al. Which patients may benefit from the use of a decision support system to improve compliance of physician decisions with clinical practice guidelines: a case study with breast cancer involving data mining. Stud Health Technol Inform. 2013;192:534-8.

42. Veerbeek L, van der Geest L, Wouters M, Guicherit O, Does-den Heijer A, Nortier J, et al. Enhancing the quality of care for patients with breast cancer: seven years of experience with a Dutch auditing system. Eur J Surg Oncol. 2011;37(8):714-8.

43. Young OE, Valassiadou K, Dixon M. A review of current practices in breast conservation surgery in the UK. Ann R Coll Surg Engl. 2007;89(2):118-23.

44. Watson GA, Deac O, Aslam R, O'Dwyer R, Tierney A, Sukor S, et al. Real-World Experience of Palbociclib-Induced Adverse Events and Compliance With Complete Blood Count Monitoring in Women With Hormone Receptor-Positive/HER2-Negative Metastatic Breast Cancer. Clinical Breast Cancer. 2019;19(1):e186-e94.

45. Bouaud J, Seroussi B. Revisiting the EBM decision model to formalize non-compliance with computerized CPGs: results in the management of breast cancer with OncoDoc2. AMIA Annu Symp Proc. 2011;2011:125-34.

46. Seroussi B, Soulet A, Messai N, Laouenan C, Mentre F, Bouaud J. Patient clinical profiles associated with physician non-compliance despite the use of a guideline-based decision support system: a case study with OncoDoc2 using data mining techniques. AMIA Annu Symp Proc. 2012;2012:828-37.

47. Seroussi B, Laouenan C, Gligorov J, Uzan S, Mentre F, Bouaud J. Which breast cancer decisions remain non-compliant with guidelines despite the use of computerised decision support? Br J Cancer. 2013;109(5):1147-56.

48. Van Ryckeghem F, Haverbeke C, Wynendaele W, Jerusalem G, Somers L, Van den Broeck A, et al. Real-world use of granulocyte colony-stimulating factor in ambulatory breast cancer patients: a cross-sectional study. Support Care Cancer. 2019;27(3):1099-108.

49. Aristei C, Amichetti M, Ciocca M, Nardone L, Bertoni F, Vidali C. Radiotherapy in Italy after conservative treatment of early breast cancer. A survey by the Italian Society of Radiation Oncology (AIRO). Tumori. 2008;94(3):333-41.

50. Barni S, Venturini M, Molino A, Donadio M, Rizzoli S, Maiello E, et al. Importance of adherence to guidelines in breast cancer clinical practice. The Italian experience (AIOM). Tumori. 2011;97(5):559-63.

51. Natoli C, Brocco D, Sperduti I, Nuzzo A, Tinari N, De Tursi M, et al. Breast cancer "tailored follow-up" in Italian oncology units: a web-based survey. PLoS One. 2014;9(4):e94063.

52. DURTO. Antiemetic prescription in Italian breast cancer patients submitted to adjuvant chemotherapy. Support Care Cancer. 2003;11(12):785-9.

53. Roila F, Ballatori E, Patoia L, Palazzo S, Veronesi A, Frassoldati A, et al. Adjuvant systemic therapies in women with breast cancer: an audit of clinical practice in Italy. Ann Oncol. 2003;14(6):843-8.

54. Ray-Coquard I, Morere JF, Scotte F, Cals L, Antoine EC. Management of anemia in advanced breast and lung cancer patients in daily practice: results of a French survey. Adv Ther. 2012;29(2):124-33.

55. Lebeau M, Mathoulin-Pelissier S, Bellera C, Tunon-de-Lara C, Daban A, Lipinski F, et al. Breast cancer care compared with clinical Guidelines: an observational study in France. BMC Public Health. 2011;11:45.

56. Liebrich C, Unger G, Dlugosch B, Hofmann S, Petry KU. Adopting Guidelines into Clinical Practice: Implementation of Trastuzumab in the Adjuvant Treatment of Breast Cancer in Lower Saxony, Germany, in 2007. Breast Care (Basel). 2011;6(1):43-50.

57. de Bock GH, Vliet Vlieland TP, Hakkeling M, Kievit J, Springer MP. GPs' management of women seeking help for familial breast cancer. Fam Pract. 1999;16(5):463-7.

58. Mylvaganam S, Conroy EJ, Williamson PR, Barnes NLP, Cutress RI, Gardiner MD, et al. Adherence to best practice consensus guidelines for implant-based breast reconstruction: Results from the iBRA national practice questionnaire survey. Eur J Surg Oncol. 2018;44(5):708-16.

59. Grandjean I, Kwast AB, de Vries H, Klaase J, Schoevers WJ, Siesling S. Evaluation of the adherence to follow-up care guidelines for women with breast cancer. Eur J Oncol Nurs. 2012;16(3):281-5.

60. Vercauteren LD, Kessels AG, van der Weijden T, Severens JL, van Engelshoven JM, Flobbe K. Association between guideline adherence and clinical outcome for patients referred for diagnostic breast imaging. Qual Saf Health Care. 2010;19(6):503-8.

61. Mille D, Roy T, Carrere MO, Ray I, Ferdjaoui N, Spath HM, et al. Economic impact of harmonizing medical practices: compliance with clinical practice guidelines in the follow-up of breast cancer in a French Comprehensive Cancer Center. J Clin Oncol. 2000;18(8):1718-24.

62. Ray-Coquard I, Philip T, Lehmann M, Fervers B, Farsi F, Chauvin F. Impact of a clinical guidelines program for breast and colon cancer in a French cancer center. Jama. 1997;278(19):1591-5.

63. Jacke CO, Albert US, Kalder M. The adherence paradox: guideline deviations contribute to the increased 5-year survival of breast cancer patients. BMC Cancer. 2015;15:734.

64. Sacerdote C, Bordon R, Pitarella S, Mano MP, Baldi I, Casella D, et al. Compliance with clinical practice guidelines for breast cancer treatment: a population-based study of quality-of-care indicators in Italy. BMC Health Serv Res. 2013;13:28.

65. Ottevanger PB, De Mulder PH, Grol RP, van Lier H, Beex LV. Adherence to the guidelines of the CCCE in the treatment of node-positive breast cancer patients. Eur J Cancer. 2004;40(2):198-204.

66. Holm-Rasmussen EV, Jensen MB, Balslev E, Kroman N, Tvedskov TF. The use of sentinel lymph node biopsy in the treatment of breast ductal carcinoma in situ: A Danish population-based study. Eur J Cancer. 2017;87:1-9.

67. Jensen MB, Laenkholm AV, Offersen BV, Christiansen P, Kroman N, Mouridsen HT, et al. The clinical database and implementation of treatment guidelines by the Danish Breast Cancer Cooperative Group in 2007-2016. Acta Oncologica. 2018;57(1):13-8.

68. Andreano AR, P; Valsecchi, MG; Russo, A. G. Adherence to guidelines and breast cancer patients survival: a population-based cohort study analyzed with a causal inference approach. Breast Cancer Res Treat. 2017;164(1):119-31.

69. Bucchi L, Foca F, Ravaioli A, Vattiato R, Balducci C, Fabbri C, et al. Receipt of adjuvant systemic therapy among patients with high-risk breast cancer detected by mammography screening. Breast Cancer Res Treat. 2009;113(3):559-66.

70. Balasubramanian SP, Murrow S, Holt S, Manifold IH, Reed MW. Audit of compliance to adjuvant chemotherapy and radiotherapy guidelines in breast cancer in a cancer network. Breast. 2003;12(2):136-41.

71. Poncet B, Colin C, Bachelot T, Jaisson-Hot I, Derain L, Magaud L, et al. Treatment of metastatic breast cancer: a large observational study on adherence to French prescribing guidelines and financial cost of the anti-HER2 antibody trastuzumab. Am J Clin Oncol. 2009;32(4):369-74.

72. Schrodi S, Niedostatek A, Werner C, Tillack A, Schubert-Fritschle G, Engel J. Is primary surgery of breast cancer patients consistent with German guidelines? Twelve-year trend of population-based clinical cancer registry data. Eur J Cancer Care (Engl). 2015;24(2):242-52.

73. Wimmer T, Ortmann O, Gerken M, Klinkhammer-Schalke M, Koelbl O, Inwald EC. Adherence to guidelines and benefit of adjuvant radiotherapy in patients with invasive breast cancer: results from a large population-based cohort study of a cancer registry. Archives of Gynecology and Obstetrics. 2019.

74. Wockel A, Varga D, Atassi Z, Kurzeder C, Wolters R, Wischnewsky M, et al. Impact of guideline conformity on breast cancer therapy: results of a 13-year retrospective cohort study. Onkologie. 2010;33(1-2):21-8.

75. Hancke K, Denkinger MD, Konig J, Kurzeder C, Wockel A, Herr D, et al. Standard treatment of female patients with breast cancer decreases substantially for women aged 70 years and older: a German clinical cohort study. Ann Oncol. 2010;21(4):748-53.

76. Varga D, Wischnewsky M, Atassi Z, Wolters R, Geyer V, Strunz K, et al. Does guideline-adherent therapy improve the outcome for early-onset breast cancer patients? Oncology. 2010;78(3-4):189-95.

77. Wockel A, Wolters R, Wiegel T, Novopashenny I, Janni W, Kreienberg R, et al. The impact of adjuvant radiotherapy on the survival of primary breast cancer patients: a retrospective multicenter cohort study of 8935 subjects. Ann Oncol. 2014;25(3):628-32.

78. Van Ewijk R, Wockel A, Gundelach T, Hancke K, Janni W, Singer S, et al. Is guideline-adherent adjuvant treatment an equal alternative for patients aged >65 who cannot participate in adjuvant clinical breast cancer trials? A retrospective multi-center cohort study of 4,142 patients. Arch Gynecol Obstet. 2015;291(3):631-40.

79. Schwentner L, Wockel A, Konig J, Janni W, Ebner F, Blettner M, et al. Adherence to treatment guidelines and survival in triple-negative breast cancer: a retrospective multi-center cohort study with 9,156 patients. BMC Cancer. 2013;13:487.

80. Ebner F, Hancke K, Blettner M, Schwentner L, Wockel A, Kreienberg R, et al. Aggressive Intrinsic Subtypes in Breast Cancer: A Predictor of Guideline Adherence in Older Patients With Breast Cancer? Clin Breast Cancer. 2015;15(4):e189-95.

81. Ebner F, van Ewijk R, Wockel A, Hancke K, Schwentner L, Fink V, et al. Tumor biology in older breast cancer patients--what is the impact on survival stratified for guideline adherence? A retrospective multi-centre cohort study of 5378 patients. Breast. 2015;24(3):256-62.

82. Wollschlager D, Meng X, Wockel A, Janni W, Kreienberg R, Blettner M, et al. Comorbidity-dependent adherence to guidelines and survival in breast cancer-Is there a role for guideline adherence in comorbid breast cancer patients? A retrospective cohort study with 2137 patients. Breast J. 2017.

83. Wolters R, Wischhusen J, Stuber T, Weiss CR, Krockberger M, Bartmann C, et al. Guidelines are advantageous, though not essential for improved survival among breast cancer patients. Breast Cancer Res Treat. 2015;152(2):357-66.

84. Wockel A, Kurzeder C, Geyer V, Novasphenny I, Wolters R, Wischnewsky M, et al. Effects of guideline adherence in primary breast cancer--a 5-year multi-center cohort study of 3976 patients. Breast. 2010;19(2):120-7.

85. Schwentner L, Wolters R, Koretz K, Wischnewsky MB, Kreienberg R, Rottscholl R, et al. Triple-negative breast cancer: the impact of guideline-adherent adjuvant treatment on survival--a retrospective multi-centre cohort study. Breast Cancer Res Treat. 2012;132(3):1073-80.

86. Schwentner L, Wolters R, Wischnewsky M, Kreienberg R, Wockel A. Survival of patients with bilateral versus unilateral breast cancer and impact of guideline adherent adjuvant treatment: a multi-centre cohort study of 5292 patients. Breast. 2012;21(2):171-7.

87. Schreuder K, Kuijer A, Rutgers EJT, Smorenburg CH, van Dalen T, Siesling S. Impact of gene-expression profiling in patients with early breast cancer when applied outside the guideline directed indication area. Eur J Cancer. 2017;84:270-7.

88. Schaapveld M, de Vries EG, van der Graaf WT, Otter R, Willemse PH. Quality of adjuvant CMF chemotherapy for node-positive primary breast cancer: a population-based study. J Cancer Res Clin Oncol. 2004;130(10):581-90.

89. Schaapveld M, de Vries EG, Otter R, de Vries J, Dolsma WV, Willemse PH. Guideline adherence for early breast cancer before and after introduction of the sentinel node biopsy. Br J Cancer. 2005;93(5):520-8.

90. de Roos MA, de Bock GH, Baas PC, de Munck L, Wiggers T, de Vries J. Compliance with guidelines is related to better local recurrence-free survival in ductal carcinoma in situ. Br J Cancer. 2005;93(10):1122-7.

91. Weggelaar I, Aben KK, Warle MC, Strobbe LJ, van Spronsen DJ. Declined guideline adherence in older breast cancer patients: a population-based study in the Netherlands. Breast J. 2011;17(3):239-45.

92. Visser A, van de Ven EM, Ruczynski LI, Blaisse RJ, van Halteren HK, Aben K, et al. Cardiac monitoring during adjuvant trastuzumab therapy: Guideline adherence in clinical practice. Acta Oncol. 2016;55(4):423-9.

93. de Munck L, Schaapveld M, Siesling S, Wesseling J, Voogd AC, Tjan-Heijnen VC, et al. Implementation of trastuzumab in conjunction with adjuvant chemotherapy in the treatment of non-metastatic breast cancer in the Netherlands. Breast Cancer Res Treat. 2011;129(1):229-33.

94. van de Water W, Bastiaannet E, Dekkers OM, de Craen AJ, Westendorp RG, Voogd AC, et al. Adherence to treatment guidelines and survival in patients with early-stage breast cancer by age at diagnosis. Br J Surg. 2012;99(6):813-20.

95. Kuijer A, Verloop J, Visser O, Sonke G, Jager A, van Gils CH, et al. The influence of socioeconomic status and ethnicity on adjuvant systemic treatment guideline adherence for early-stage breast cancer in the Netherlands. Ann Oncol. 2017;28(8):1970-8.

96. Heins MJ, de Jong JD, Spronk I, Ho VKY, Brink M, Korevaar JC. Adherence to cancer treatment guidelines: influence of general and cancer-specific guideline characteristics. European journal of public health. 2017;27(4):616-20.

97. Schwentner L, Van Ewijk R, Kuhn T, Flock F, Felberbaum R, Blettner M, et al. Exploring patient- and physician-related factors preventing breast cancer patients from guideline-adherent adjuvant chemotherapy-results from the prospective multi-center study BRENDA II. Support Care Cancer. 2016;24(6):2759-66.

98. Stuber T, van Ewijk R, Diessner J, Kuhn T, Flock F, Felberbaum R, et al. Which patient- and physician-related factors are associated with guideline adherent initiation of adjuvant endocrine therapy? Results of the prospective multi-centre cohort study BRENDA II. Breast Cancer. 2017;24(2):281-7.

99. Leinert E, Schwentner L, Blettner M, Wockel A, Felberbaum R, Flock F, et al. Association between cognitive impairment and guideline adherence for application of chemotherapy in older patients with breast cancer: Results from the prospective multicenter BRENDA II study. Breast Journal. 2019.

100. Boskovic L, Gasparic M, Petkovic M, Gugic D, Lovasic IB, Soldic Z, et al. Bone health and adherence to vitamin D and calcium therapy in early breast cancer patients on endocrine therapy with aromatase inhibitors. Breast. 2017;31:16-9.

101. Smith SG, Side L, Meisel SF, Horne R, Cuzick J, Wardle J. Clinician-Reported Barriers to Implementing Breast Cancer Chemoprevention in the UK: A Qualitative Investigation. Public Health Genomics. 2016;19(4):239-49.

102. Andreano A, Rebora P, Valsecchi MG, Russo AG. Adherence to guidelines and breast cancer patients survival: a population-based cohort study analyzed with a causal inference approach. Breast Cancer Res Treat. 2017;164(1):119-31.
